# Microscopic basis of reaction center modulation in PsbA variants of photosystem II

**DOI:** 10.1073/pnas.2417963122

**Published:** 2025-05-12

**Authors:** Sinjini Bhattacharjee, Igor Gordiy, Abhishek Sirohiwal, Dimitrios A. Pantazis

**Affiliations:** ^a^Department of Molecular Theory and Spectroscopy, Max-Planck-Institut für Kohlenforschung, Mülheim an der Ruhr 45470, Germany; ^b^Department of Inorganic and Physical Chemistry, Division of Chemical Sciences, Indian Institute of Science, Bangalore 560012, India

**Keywords:** photosynthesis, charge transfer, excited states, isoforms, multiscale simulations

## Abstract

Photosystem II (PSII) is a protein–pigment complex that utilizes sunlight to catalyze water oxidation and plastoquinone reduction, initiating the electron transfer (ET) cascade in oxygenic photosynthesis. The D1 and D2 proteins are the most important transmembrane subunits of PSII that bind all redox-active components involved in primary charge separation (CS) and ET. D1 is susceptible to oxidative photodamage, particularly under high light, and protection partly involves genetic regulation. Cyanobacterial D1 is encoded by the *psbA* gene family that expresses distinct isoforms (PsbA1–3) depending on environmental conditions. Most differences in D1 isoforms are close to the active-branch reaction center (RC) pigments P_D1_, P_D2_, Chl_D1_, and Pheo_D1_. Here, we combine molecular dynamics simulations with multiscale quantum-mechanics/molecular-mechanics calculations on the membrane-bound PSII monomer of each variant to compare the redox and excited state properties of RC pigments using long-range-corrected density functional theory. We identify specific amino acid substitutions responsible for electrochromic shifts on distinct pigments and pigment groups. Our results indicate that the Pheo_D1_ acceptor is the primary regulatory target. The redox properties of the Chl_D1_–Pheo_D1_ pair and the energetics of Chl_D1_^δ+^Pheo_D1_^δ-^ charge-transfer states are distinctly modulated in the three isoforms: Compared to the standard *psbA*_1_, charge separation is inhibited in *psbA*_2_ and facilitated in *psbA*_3_ PSII. The results provide a microscopic description of how genetic variations modulate protein electrostatics and influence primary processes in photosynthetic reaction centers.

## Significance

The arrangement of macrocyclic pigments within the evolutionarily optimized electrostatic environment of membrane-embedded proteins underlies the function of reaction centers, the sites where the harvested energy of sunlight initiates the electron flow in oxygenic photosynthesis. There are two ways to modulate the physicochemical properties of reaction centers. The first involves substituting a pigment of a certain chemical type with another. The second involves changing the matrix by expressing an alternate isoform of the host protein. This study employs multiscale simulations to explore how three genetic variants of a key reaction center protein in photosystem II influence the optical and redox properties of the pigments critical to the primary processes of oxygenic photosynthesis.

**Fig. 1.**
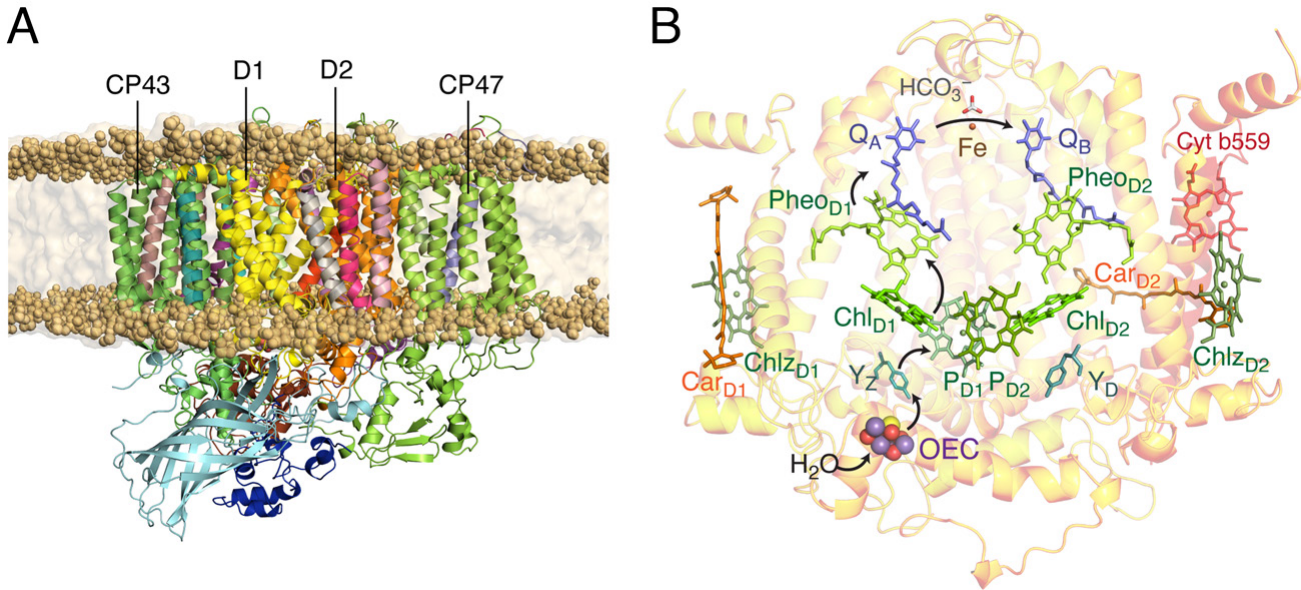
(*A*) Structure of the membrane-embedded PSII monomer. (*B*) The reaction center (RC) cofactors oriented with respect to the D1 and D2 proteins; arrows indicate the flow of electrons.

Photosystem II (PSII) of oxygenic photosynthesis is a dimeric multisubunit protein–pigment complex responsible for the four-electron oxidation of water into molecular oxygen and the two-electron reduction of a mobile plastoquinone (1–6). Cyanobacterial PSII comprises 17 transmembrane and 3 extrinsic proteins (1, 7–12). The core complex of PSII consists of the six large proteins D1 (PsbA), D2 (PsbD), CP43 (PsbC), CP47 (PsbB)(13) and Cyt_b559_ (PsbE, PsbF) (14, 15). D1 and D2 bind most active components of the electron transfer chain including the reaction center (RC) pigments and the oxygen-evolving complex (OEC) (16). The RC is the site of the light-driven charge separation and primary electron transfer. It consists of four chlorophylls, namely the P_D1_P_D2_ central pair flanked by Chl_D1_ and Chl_D2_, and two pheophytins Pheo_D1_ and Pheo_D2_, arranged pseudosymmetrically along the D1 and D2 heterodimer (Fig. 1). Excitation energy transfer from external light harvesting complexes (17–19) and the core antennae (20) CP43 and CP47 triggers primary charge separation along the D1 branch of the RC (17, 21, 22).

The D1 protein, encoded by the *psbA* gene family (23), undergoes light-induced turnover to protect PSII from photodamage (24–26). In higher plants and green algae, there is a single *psbA* gene (27) but cyanobacteria may possess multiple copies (27–33), whose expression occurs in response to environmental factors such as high light, UV light, or varying temperature (27, 29, 34, 35). In the mesophilic cyanobacterium *Synechocystis* PCC 6803, D1 is encoded by three variants (*psbA*_1_*, psbA*_2_, and *psbA*_3_), among which *psbA_*2*_* and *psbA_*3*_* encode the same D1 isoform expressed under various stress conditions, whereas *psbA_*1*_* encodes a distinct isoform. *Synechococcus* PCC 7942 also has three *psbA* genes that encode two different D1 isoforms, D1:1 by *psbA*_1_ and D1:2 by *psbA*_2_ and *psbA*_3_. In the thermophilic cyanobacterium *T. elongatus*, three *psbA* genes have also been identified, encoding three distinct D1 isoforms: *psbA*_1_ is expressed under normal growth, *psbA*_3_ at high light (30, 36, 37) and *psbA*_2_ is partially activated under microaerobic conditions. Despite insights from site-directed mutagenesis combined with spectroscopy or with crystallographic analysis of mutant strains (28–30, 36–40), a microscopic understanding of how the different isoforms regulate the properties and function of the RC remains elusive.

Under physiological conditions, charge-transfer (CT) excited states of Chl_D1_*^δ^*^+^Pheo_D1_*^δ^*^–^ character are created in the PSII-RC (17, 41, 42) presumably leading to formation of a transient charge-separated (CS) state in the form of Chl_D1_^+^Pheo_D1_^–^ (43–48) (we stress that the term “CT states” refers to vertical excited states whereas the term “CS states” refers to conformationally relaxed metastable radical pair states) before the electron hole delocalizes onto P_D1_P_D2_ to form the P_680_^+^Pheo_D1_^–^ radical pair (49, 50). P_680_^+^ is the highly oxidizing radical cation (*E*_m_ ~1.1 to 1.3 V) that drives water oxidation (46, 51). Protein matrix electrostatics are the principal factor for differentiating the properties of otherwise chemically identical pigments, generating functional asymmetry in the RC and enabling formation of interpigment CT states of Chl_D1_*^δ^*^+^Pheo_D1_*^δ^*^–^ or P_D1_*^δ^*^+^Pheo_D1_*^δ^*^–^ character that can act as precursors to the primary charge separated radical pairs (52). Given that each *psbA* variant has specific sequence differences, the RC pigments are embedded in a slightly different protein environment in each case. It is therefore essential to understand how these differences influence the excitation profile, redox properties, and primary CT states of RC pigments. Here, we combine large-scale molecular dynamics (MD) simulations with multilevel quantum-mechanics/molecular-mechanics (QM/MM) calculations on membrane-bound PSII monomer models for the three PsbA-PSII variants. We compare the redox and optical properties of the critical Chl_D1_–Pheo_D1_ pair using long-range corrected time-dependent density functional theory (TD-DFT) and identify residues responsible for specific matrix-induced adjustments of the electronically excited CT states and redox properties in this crucial pigment pair.

## Results

### Structural Analysis of PsbA Variants.

**Fig. 2.**
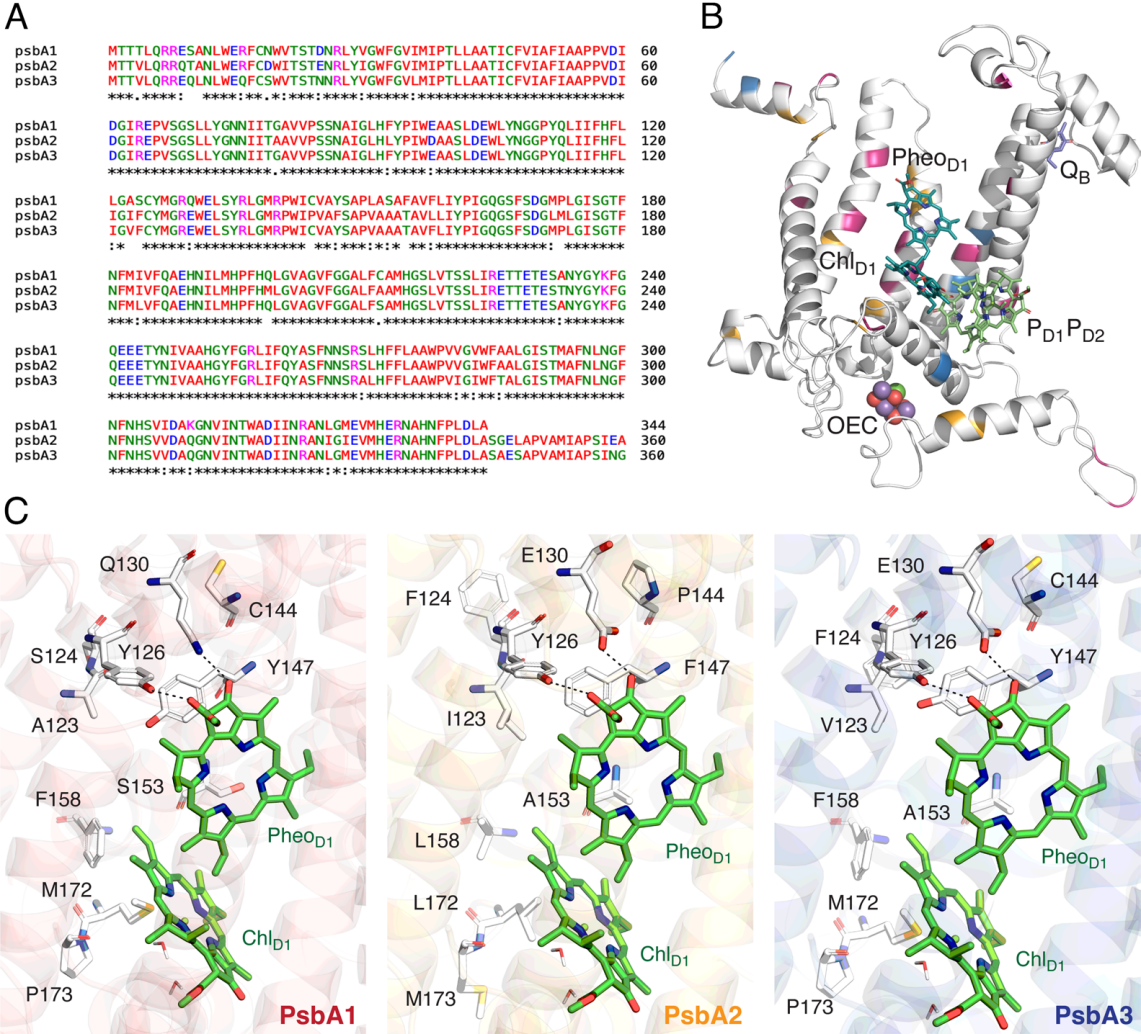
(*A*) Sequences of the three PsbA proteins (A1: *T. vulcanus,* A2 and A3: *T. elongatus)* compared using Clustal Omega. (*B*) Schematic of locations of D1 substitutions compared to PsbA1 along with selected cofactors (Chl_D1_, Pheo_D1_, P_D1_–P_D2_, OEC, and Q_B_): Residues that differ only in PsbA2 are marked in orange, PsbA3 in blue, and both in PsbA2/A3 are pink. (*C*) Comparison of D1 protein substitutions around the Chl_D1_–Pheo_D1_ pair among the three PsbA variants based on QM/MM optimized structures in PsbA1–A3. E130 is protonated and participates in direct hydrogen bonding with Pheo_D1_ in PsbA2 and A3. Specific hydrogen bonding interactions are indicated with dashed lines.

The folded mature D1 protein in thermophilic cyanobacteria contains 344 residues within five transmembrane helices (TMH A–E) in each of the three PsbA variants (28, 31). Sequence analysis shows 21 substitutions between PsbA1/PsbA3, 31 between PsbA1/PsbA2, and 27 between PsbA2/PsbA3, indicating that all three variants are highly conserved (Fig. 2*A*).

Segments with most significant variations are the N-terminus region (1–30) with 10 variant residues, followed by six variations in the TMH-C residues (144–158) in the vicinity of the redox-active Tyr161 (Y_Z_). The nature and location of most PsbA substitutions (Fig. 2*B*) support the proposed influence on spectroscopic and redox properties of the RC. Substitutions located near RC cofactors participating in charge separation and electron transfer compose 64% (18 out of 28) of differences between PsbA1/PsbA2 and 57% (12 out of 21) between PsbA1/PsbA3. Substitutions located more than 15 Å away from any RC cofactor (*SI Appendix*, Table S1) are not expected to influence optical and redox properties, therefore subsequent analysis is focused on substitutions closest to active branch pigments. A general trend observed for these substitutions is that PsbA1 residues with polarizable side chain heteroatoms (sulfur or oxygen) are often substituted by aliphatic hydrophobic residues (e.g., M172L and M328I in PsbA2, and S153A, S270A in PsbA3).

Focusing on the donor side of PSII, the substitution at D1-172 is closest to the primary donor Chl_D1_. In PsbA1 and PsbA3 PSII structures (28) the M172 side chain is oriented toward the macrocyclic ring of Chl_D1_ at a distance of 3.8 Å (28). The PsbA2 variant has a leucine at this position slightly further at 4.2 Å. Only the D1-286 residue differs in PsbA1 sequences of *T. elongatus* and *T. vulcanus*. In the former, it is a threonine (T286) H-bonded to the acetyl moiety of P_D1_, while the latter has a hydrophobic alanine. A286 is conserved in PsbA2 of *T. elongatus* while T286 is found in the PsbA3 variant. In sequences where T286 is replaced by A286, a water is H-bonded to the acetyl group of P_D1_ suggesting that this substitution may destabilize P_D1_ due to the loss of a H-bonding interaction (39). The D1-P173M substitution in PsbA2 is also shown to contribute toward structural differences compared to PsbA1/A3 (28). Particularly, recent crystallographic studies suggested that this substitution can lead to the loss of two water molecules due to narrowing of the Cl-1 channel of the OEC (28), but further investigations are required to understand the functional implications of this difference.

On the acceptor side, two important substitutions are found in the binding pocket of Pheo_D1_. In PsbA1 the two closest substitution sites are Q130, which is H-bonded to the 13^1^-keto group of Pheo_D1_, and D1-Y147 that acts as H-bonding partner to the C = O of the ester group. Based on crystal structures and sequence analysis (28, 31) Q130 of PsbA1 is replaced by a glutamate (E130) in PsbA2 and PsbA3, whereas Y147 is replaced by a nonpolar F147 in PsbA2. Pheo_D1_ has a third H-bonding partner (Y126) to the acetyl group, but this residue remains invariant across the three isoforms. The only substitution that occurs at the Q_B_ pocket is at D1-270, which is serine in PsbA1/A2 but alanine in PsbA3. This was proposed to result in loss of an H-bond between Ser270 and the sulfoquinovosyl-diacylglycerol (SQDG) lipid near Q_B_, which affects the binding properties of Q_B_ and the exchange efficiency of Q_B_H_2_ (28, 53). Further important substitutions are at site 212 where C212 in PsbA1 is substituted for alanine in PsbA2 and serine in PsbA3. This site is centrally located between the D1/D2 branches close to Pheo_D2_ but equidistant from nonheme Fe, Q_A_, Q_B_, and Pheo_D1_ and may have an electrostatic effect on secondary ET processes in the D2 side.

Overall, the differences between PsbA variants are expected to have the greatest influence on P_D1_, Chl_D1_, Pheo_D1_, and the OEC, but proximity analysis suggests that the differences in each variant are mostly relevant for Chl_D1_ and Pheo_D1_, while they are farthest from the OEC and P_D1_P_D2_. Therefore, the latter cofactors do not seem to be targeted in the genetic variants and subsequent analysis in this work is focused on the properties of the Chl_D1_–Pheo_D1_ pair.

### Functional Dynamics Around Pheo_D1_.

Large-scale MD simulations enable us to obtain a realistic view of the immediate environment of RC pigments and to extensively sample the global conformational changes of the protein matrix. Analysis of MD trajectories indicates that the D1 polypeptides are relatively more rigid throughout the simulations compared to other components of PSII (*SI Appendix*, Fig. S2) for all PsbA variants. Based on relative RMSD (*SI Appendix*, Fig. S2) and structural comparisons, we confirm that the three variants do not undergo large-scale conformational changes within the MD timescales. The relative orientations of RC pigments also remain invariant (*SI Appendix*, Table S2).

Crystallographic studies reported RMSD of 0.27 Å for the C_α_ atoms between PsbA1/PsbA2-PSII, 0.25 Å for PsbA1/PsbA3-PSII, and 0.20 Å for PsbA2/PsbA3-PSII. In the current study, a comparative RMSD analysis of the whole PSII protein confirms that the multiple changes in the PsbA2 and PsbA3 variants have very limited effect on the overall protein structure.

Recent structural studies (28) revealed that Pheo_D1_ in PsbA2-PSII has a longer H-bonding distance with D1-Y126, while the D1-Y147F substitution eliminates another H-bond that is present in PsbA1 and PsbA3, implying that Pheo_D1_ might be less strongly bound in PsbA2. In PsbA1/A3, Y147 acts as a direct H-bonding partner to the ester C = O group and stabilizes Pheo_D1_ by 4.5 kcal mol^–1^ in PsbA1 and A3, whereas in PsbA2 the hydrophobic F147 leads to a loss of this crucial H-bonding interaction, slightly destabilizing Pheo_D1_. Molecular Mechanics Poisson–Boltzmann Surface Area (MM-PBSA) (54) calculations reveal that with the exception of D1-130 and D1-147, all residues that contribute to the stability of Pheo_D1_ are conserved across the three variants. Based on calculated binding energies, we find that indeed the Pheo_D1_ in PsbA2-PSII (∆*G*_binding_ = −78.37 kcal mol^–1^) is less strongly bound compared to PsbA1 (−81.76 kcal mol^–1^) and PsbA3 (−81.81 kcal mol^–1^). The energy components and residue-wise decomposition of the total binding energy of Pheo_D1_ for each model is depicted in *SI Appendix*, Table S3 and *SI Appendix*, Fig. S3.

### Redox Properties of the Chl_D1_–Pheo_D1_ Pair.

The properties of RC pigments are influenced by various factors such as H-bonding, axial ligation, macrocyclic ring curvature, out-of-plane motion of π-conjugated groups, electronic couplings (55–62), and protein electrostatics (41, 42, 52, 63, 64). However, the primary events in PSII are also correlated to extrinsic parameters like the wavelength and intensity of incident radiation. Far-red light-acclimated cyanobacteria (29, 65–72) adapt by substituting different chlorophylls with red-shifted variants (Chl *d*, *f*) to utilize longer wavelengths efficiently. On the other hand, the PsbA variants in thermophilic cyanobacteria illustrate how changes in the protein matrix modulate RC function, given that charge separation is enabled through matrix electrostatics (41).

Substitutions that cause variations in the H-bonding environment around the Chl_D1_–Pheo_D1_ pair directly tune the Pheo_D1_/Pheo_D1_^–^ redox potential (E_m_) (30, 36, 38, 39, 43, 73–75). A detailed analysis of the frontier molecular orbitals of the Chl_D1_–Pheo_D1_ pair can explain the electronic origin of the *E*_m_ shifts (Pheo_D1_^–^/Pheo_D1_) for each D1 variant. We estimated the vertical electron affinity (EA) of Pheo_D1_ by the electronic energy difference of the Pheo_D1_^–^/Pheo_D1_ redox couple by QM(DFT)/MM. A comparison of the Pheo_D1_/Pheo_D1_^–^ EAs across an ensemble of protein configurations (*SI Appendix*, Fig. S4*A*) suggests that Pheo_D1_ in PsbA3 (0.70 ± 0.36 eV) has the highest average EA followed by PsbA1 (0.52 ± 0.32 eV) and PsbA2 (0.51 ± 0.30 eV). This trend is consistent across most configurations implying that the electron-accepting tendency of Pheo_D1_ is highest in PsbA3, which causes its *E*_m_ to be more positive compared to that in PsbA1/PsbA2. This is opposite to what is reported for PsbA2 even though both PsbA2/A3 possess the same Pheo_D1_–E130 interaction. This demonstrates that the shift in the *E*_m_ (Pheo_D1_^–^/Pheo_D1_) cannot be explained based on a single Q130E substitution, but is instead a cumulative effect. We also computed the ionization energies (IEs) of Chl_D1_ from the HOMO energies of the Chl_D1_–Pheo_D1_ pair applying Koopmans’ theorem and find that IE values also vary among the different variants (*SI Appendix*, Fig. S4*B*) (PsbA1: 3.15 ± 0.41 eV, PsbA2: 3.54 ± 0.31 eV, PsbA3: 3.32 ± 0.35 eV). Chl_D1_ is therefore the easiest to oxidize in PsbA1, followed by PsbA3 and PsbA2. Furthermore, based on the relative differences between the IE of Chl_D1_^+^/Chl_D1_ and EA of Pheo_D1_^–^/Pheo_D1_, as shown in *SI Appendix*, Fig. S4*C*, the vertical energy difference between the unrelaxed Chl_D1_^+^Pheo_D1_^–^ state and neutral Chl_D1_Pheo_D1_ state is found to be lowest in PsbA3 (2.64 ± 0.18 eV) followed by PsbA1 (2.89 ± 0.15 eV) and PsbA2 (3.07 ± 0.16 eV).

In order to estimate free energies in the presence of explicit PSII protein electrostatics and account for dynamic changes, we additionally employed the Perturbed Matrix Method (PMM) with MD trajectories (76). The overall free energy change (∆*G*) for the formation of Chl_D1_^+^Pheo_D1_^–^ from Chl_D1_Pheo_D1_ is calculated to be –0.88 eV for PsbA2-Glu(H), –1.04 eV for PsbA1 and –1.32 eV for PsbA3-Glu(H), respectively. We note that the SD in the calculated IP–EA differences and the uncertainties in the PMM free energy differences are higher than the experimentally deduced *E*_m_ differences (17 to 35 mV) in the PsbA variants. Therefore, the above values can be seen as preliminary results indicating trends, but not yet as the basis for quantitative analysis. A thorough benchmarking of QM/MM methods and alternative sampling methodologies utilizing long MD trajectories will need to be pursued before more precise *E*_m_ value predictions can be achieved.

### Global Tuning of Chl_D1_–Pheo_D1_ Excited States.

Vertical excitation energies for the Chl_D1_–Pheo_D1_ pair were computed using QM(TD-DFT)/MM. The QM region consisted of the two pigments with axial waters and the immediate H-bonded residues (Fig. 2*C*). The nature of excited states was established using Natural Transition Orbitals (NTOs) (Fig. 4*A* and *SI Appendix*, Table S4), which are the orbitals that diagonalize the transition density and offer the most compact and chemically transparent description of each excitation. NTO coefficients indicate that the lowest excited states (S_1_ and S_2_) possess variable extent of Chl_D1_*^δ^*^+^Pheo_D1_*^δ^*^–^ CT character in all PsbA variants, and the distribution of the CT character along the MD trajectory is determined by the protein conformation and pigment geometries. The lowest excited state with significant Chl_D1_*^δ^*^+^Pheo_D1_*^δ^*^–^ CT character within the “crystal-like” snapshot lies at 1.885 eV for PsbA1, 1.932 eV for PsbA2 and 1.701 eV for PsbA3 (*SI Appendix*, Fig. S5 and Table S5).

The excitation energies of P_D1_–P_D2_ were also computed to examine whether primary charge separation pathways differ among the D1 isoforms, for instance, if P_D1_ can act as primary donor to yield P_D1_^+^P_D2_^–^ or P_D1_^+^Chl_D1_^–^ states. In the case of PsbA1-PSII, it has been shown that the Chl_D1_ pathway is dominant, whereas the excitation profile of P_D1_–P_D2_ mainly consists of superpositions of individual local excitations with the lowest P_D1_*^δ^*^+^P_D2_*^δ^*^–^ CT state as high as 3 eV. Our present results on PsbA2 and PsbA3 are consistent with the findings on PsbA1 and provide a similar picture for the P_D1_–P_D2_ pair (*SI Appendix*, Fig. S6 and Table S6). The lowest P_D1_*^δ^*^+^P_D2_*^δ^*^–^ CT state is computed at 3.03 eV for PsbA2 and 2.87 eV for PsbA3, confirming that the central pair has no role in primary charge separation in any of the three variants. The S_1_ state in PsbA1, has an equal contribution from P_D1_ and P_D2_, while that of PsbA2 and A3 shows a dominant contribution from P_D2_ and P_D1_, respectively (*SI Appendix*, Fig. S6). Overall, our results rule out the possibility of a low-energy CT state within P_D1_–P_D2_ and suggest that PsbA substitutions do not change the site of initial CS in PSII.

The results remain robust when considering protein dynamics. TD-DFT calculations were performed on optimized QM/MM structures of the Chl_D1_–Pheo_D1_ pair on an ensemble of 65 distinct snapshots chosen from independent MD simulations of the PSII–membrane complex. This has the advantage that excited states are computed on uncorrelated protein configurations which are properly hydrated and equilibrated with the protein and lipid bilayer at the same time. The relative trends in excited state energetics for each variant are summarized in Fig. 4*B* and *SI Appendix*, Figs. S7 and S8. Focusing on Chl_D1_–Pheo_D1_, we find that the distribution of the lowest CT state varies with respect to protein dynamics in each case. The overall trends indicate that Chl_D1_*^δ^*^+^Pheo_D1_*^δ^*^–^ CT states are slightly lower in energy in PsbA3 (1.76 ± 0.21 eV), compared to that in PsbA1 (1.96 ± 0.18 eV) and PsbA2 (2.00 ± 0.17 eV). Moreover, the probability of the Chl_D1_*^δ^*^+^Pheo_D1_*^δ^*^–^ CT being dominant in the lowest excited state (S_1_) of the dimer, was much higher in the case of PsbA3 (~45% snapshots) than for PsbA1 (~25%). Overall, our results indicate that the Chl_D1_*^δ^*^+^Pheo_D1_*^δ^*^–^ CT states are more accessible in PsbA3 than PsbA1 and PsbA2.

### Specific Effects of D1 Substitutions.

The residue at D1-130 has been a major target for mutagenesis and the Q130E mutation in PsbA3 is reported to shift *E*_m_ (Pheo_D1_^–^/Pheo_D1_) by 30 to 35 meV and partially modulate the energy of the Chl_D1_^+^Pheo_D1_^–^ radical pair (77). Similar findings were observed in D1-Q130E mutants of *Synechocystis* PCC 6803 (78–80), and the corresponding D1-E130Q substitution in *Chlamydomonas reinhardtii* (79, 81). On the other hand, thermoluminescence and fluorescence studies of *T. elongatus* with PsbA1 and PsbA3 PSII showed that multiple amino acid substitutions had significantly less impact on S_2_Q_A_^–^ charge recombination compared to a single mutation at D1-130 (80, 82, 83). This raises two important questions: (i) How does a single mutation at D1-130 affect the optical properties of the Chl_D1_–Pheo_D1_ pair? and (ii) Do other D1 substitutions have compensatory effects and if so, which residues are responsible?

The first objective led us to construct independent QM/MM models for three “mutants” i.e., PsbA1-Q130E, PsbA2/PsbA3-E130Q, where only the D1-130 residue (Gln/Glu(H)) was substituted compared with the respective wild-type (WT) composition. Interestingly, our calculations on the D1-130 mutants reveal that the effect of the point mutation differs for each D1 isoform (*SI Appendix*, Fig. S9). In PsbA1-Q130E, the local excitations on Chl_D1_–Pheo_D1_ are unaffected, but the lowest Chl_D1_*^δ^*^+^Pheo_D1_*^δ^*^–^ CT state is blue-shifted by 0.024 eV (194 cm^-1^) compared to the WT. In PsbA2 the CT state remains invariant to the E130Q mutation, whereas in PsbA3-PSII the same mutation blue-shifts the CT states. It is important to identify other key residues affecting electrochromic shifts in the optical absorption spectra of Chl_D1_–Pheo_D1_, especially spectral tuning of the Chl_D1_*^δ^*^+^Pheo_D1_*^δ^*^–^ CT states, therefore we selected all naturally occurring substitutions close to the Chl_D1_–Pheo_D1_ pair (10 Å) involving a substitution to a polar residue in PsbA2/A3 compared to PsbA1 (Table 1). The substitutions investigated are at D1-130, D1-144, D1-147, D1-153, D1-158, D1-172, and D1-212.

**Table 1.** Location and identity of all variant residues within the copies of the PsbA1 protein from *T. vulcanus*, and the PsbA2 and PsbA3 proteins from *T. elongatus*. Residues within 15 Å from any cofactor of the RC are listed

Position	A1	A2	A3	Nearest cofactor	A1	A2	A3
123	Ala	Ile	Val	Chl_D1_/Pheo_D1_	8/9	6.1/7.7	6.9/7.8
124	Ser	Phe	Phe	Pheo_D1_	13.1	12.8	12.8
**144**	Cys	Pro	Cys	Pheo_D1_	7.8	6.3	7.8
80	Gly	Ala	Gly	Chl_D1_	10.6	10.5	10.8
173	Pro	Met	Pro	Chl_D1_/P_D1_/OEC	8.1/9.9/9.4	9.1/9.9/7.1	8.0/9.8/9.3
199	Gln	Met	Gln	P_D1_/Chl_D2_	9.0/3.6	8.9/3.6	9.3/3.4
121	Leu	Ile	Ile	Chl_D1_/Pheo_D1_	13.7/14.9	13.7/14.3	13.6/14.4
**130**	Gln	Glu	Glu	Pheo_D1_/Q_A_	2.9/9.3	2.7/9.2	2.7/9.6
**147**	Tyr	Phe	Tyr	Pheo_D1_	3.4	3.6	3.7
151	Leu	Val	Val	Pheo_D1_/Chl_D1_	8.6/8.8	7.8/9.2	7.3/9
**153**	Ser	Ala	Ala	P_D1_/Chl_D1_/Pheo_D1_	3.7/5.4/7.2	4.0/5.3/8.0	3.8/5.8/8.1
**158**	Phe	Leu	Phe	Chl_D1_/P_D1_/Pheo_D1_	4.1/9.1/8.9	5.4/9.9/10.7	3.8/9.3/9.0
**172**	Met	Leu	Met	P_D1_/Chl_D1_/OEC	5.7/3.8/11.0	6.8/4.2/11.3	5.8/3.8/11.0
184	Ile	Ile	Leu	P_D1_/P_D2_/OEC	4.2/6.4/10.4	4.2/6.6/10.7	4.0/5.9/10.3
**212**	Cys	Ala	Ser	P_D2_/Pheo_D2_/Pheo_D1_/Q_A_/Fe/Q_B_	12.6/6.5/9.7/10.4/10.1/10.0	12.8/7.1/9.7/11.0/9.6/9.2	12.8/7.1/9.9/10.4/9.6/9.7
270	Ser	Ser	Ala	Fe/Q_B_	12.1/8.5	12.1/8.9	11.0/8.4
281	Val	Val	Ile	P_D1_/Pheo_D1_	11.6/8.9	11.6/8.7	11.9/8.8
283	Val	Ile	Ile	P_D1_/Chl_D1_/Pheo_D1_	5.1/8.6/3.8	4.1/8.4/3.8	4.0/8.9/3.8
286	Ala	Ala	Thr	P_D1_/Chl_D1_/Pheo_D1_	3.4/8.3/8.1	3.4/8.2/8.0	3.6/7.9/7.8
326	Leu	Ile	Leu	OEC	12.7	12.8	12.2
328	Met	Ile	Met	P_D1_/P_D2_/OEC	5.4/7.9/9.6	6.8/9.7/8.4	5.3/8.8/9.4

All residues belong to the D1 subunit of the PSII monomer. The values represent the distance between the closest sidechain atom from a heavy atom (i.e., nonhydrogen atom) of the respective cofactor in the equilibrated MD snapshot for each variant.

The electrostatic contribution of each residue to the electrochromic shifts for the Chl_D1_–Pheo_D1_ pair is provided in Fig. 5. In PsbA1-PSII the exciton on Chl_D1_ is most red-shifted by S153 (−97 cm^−1^), M172 (−73 cm^−1^), and F158 (−40 cm^−1^) while Pheo_D1_ is blue-shifted by Y147 (105 cm^−1^) and Q130 (97 cm^−1^). Interestingly, both Q130 and S153 stabilize the Chl_D1_*^δ^*^+^Pheo_D1_*^δ^*^–^ states to a large extent, with minor contributions from Y147 (Fig. 5). D1-M172 and F158 are conserved in PsbA3 but substituted by L172 and L158, respectively, in PsbA2 (Table 1). In PsbA2-PSII, A153 lowers the Chl_D1_ excitation energy (−64 cm^−1^), while E130 (210 cm^−1^) and F147 (73 cm^−1^) blue-shifts Pheo_D1_. These findings demonstrate that even subtle changes in the H-bonding strength and hydrophobic environment in the D1-Q130E and D1-Y147F substitutions directly modulate the optical properties of Pheo_D1_ in PsbA2 compared to PsbA1. The more pronounced effect is seen in the Chl_D1_*^δ^*^+^Pheo_D1_*^δ^*^–^ CT states, where all three residues E130, F147, and A153 red-shift the excitation energy. Consequently, one would expect that the Chl_D1_*^δ^*^+^Pheo_D1_*^δ^*^–^ CT state in PsbA2 would be stabilized to a similar extent as that of PsbA1. However, our results (Fig. 5) indicate that the Chl_D1_*^δ^*^+^Pheo_D1_*^δ^*^–^ CT state in PsbA2 (1.932 eV) is instead blue-shifted compared to PsbA1 (1.885 eV) because of the additional D1-M172L and D1-C144P substitutions close to Chl_D1_. The influence of the D1 substitutions is most pronounced in PsbA3-PSII where A153 and M172 is found to red-shift the energy of Chl_D1_ whereas E130, Y147 both red-shifts Chl_D1_ and blue shifts Pheo_D1_. The energetics of CT states are also drastically affected by the PsbA1/A3 substitutions, with E130 contributing the most (581 cm^−1^) followed by A153 (436 cm^−1^) and Y147 (411 cm^−1^). The overall electrostatic potential (ESP) experienced by the Chl_D1_–Pheo_D1_ pair in the D1 matrix also slightly differs in each variant (*SI Appendix*, Fig. S11). Pheo_D1_ resides in a relatively more positive ESP pocket in PsbA2/A3 whereas the Chl_D1_ pocket in PsbA1/A3 is more electronegative. Therefore, the native protein matrix electrostatics in PsbA3 favor formation of the Chl_D1_*^δ^*^+^Pheo_D1_*^δ^*^–^ state more than in PsbA1/A2.

## Discussion

### Structural Changes Around Chl_D1_–Pheo_D1_.

**Fig. 3.**
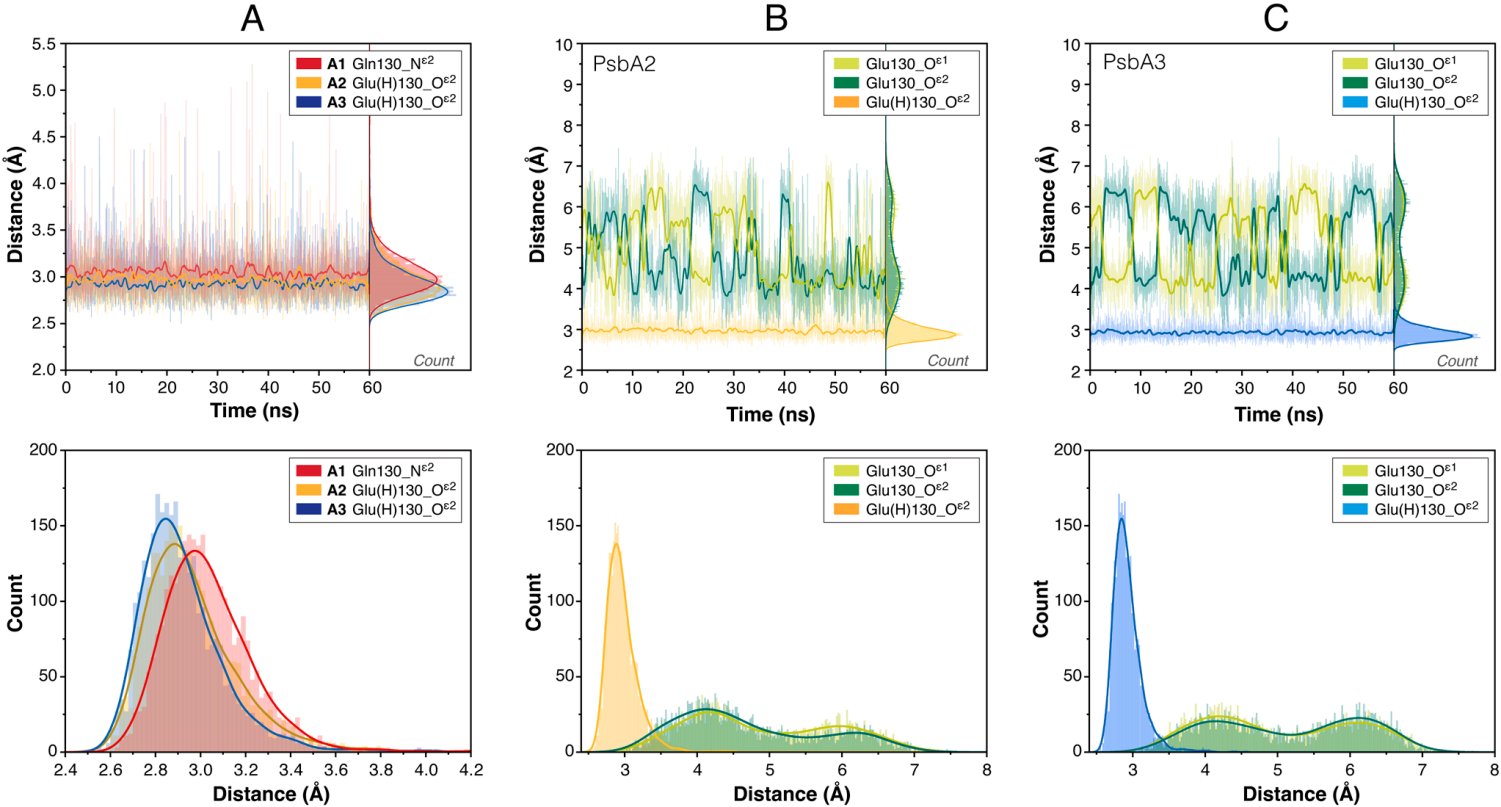
(*A*) H-bonding distances between the Pheo_D1_ carbonyl group and sidechain atoms of D1-130 along the MD trajectory for PsbA1 (red), PsbA2 (yellow), and PsbA3 (blue); (*B*) distances between the Pheo_D1_ carbonyl group and sidechain oxygen atoms of deprotonated and protonated Glu130 in PsbA2; (*C*) similarly for PsbA3.

The D1-Q130E substitution in cyanobacterial PSII-RC has been extensively discussed (30, 37, 39, 73). Site-directed mutagenesis and recent crystallographic studies showed that the D1-130 residue (Q130E) is 2.7 to 2.8 Å from the 13^1^ C = O group of Pheo_D1_, indicating a noncovalent interaction. This important structural difference is due to the change in the H-bonding environment of Pheo_D1_ caused by the substitution. Despite varying H-bonding partners, the interaction between Pheo_D1_ and the PSII protein matrix at this position is conserved across all variants. Our simulations raise a critical question regarding the protonation of E130 in PsbA2 and PsbA3-PSII. Based on the evolution (Fig. 3) of H-bonding distances between D1-130 and the 13^1^-keto group of Pheo_D1_, we find that the H-bond between Pheo_D1_ and D1-130 is shorter in PsbA2- and PsbA3- PSII due to the Q130E change, requiring E130 to be protonated as glutamic acid Glu(H). Mutagenesis studies employing Raman (84), EPR (85), and FTIR spectroscopy (30, 73) proposed that the E130 side chain acts as a H-bond donor supporting the protonation of D1-E130. In our models where the E130 in PsbA2/A3 is deprotonated (*SI Appendix*, Fig. S12), the distance between D1-E130 and Pheo_D1_ fluctuates significantly within the MD timescales as the carboxylate group’s flexibility does not facilitate a H-bond with Pheo_D1_. The relative trends in the total binding energy of Pheo_D1_ in each model also support the presence of Glu(H). Based on the MM-PBSA analysis, the presence of a charged glutamate in the vicinity destabilizes Pheo_D1_ by 7.5 kcal mol^−1^ in PsbA2 and 7.8 kcal mol^−1^ in PsbA3-PSII, compared to Glu(H). Furthermore, a charged residue at this site is unexpected since lipid bilayer regions are typically hydrophobic. In addition, the number of water molecules and H-bonds within a 5 Å radius are conserved, suggesting that E130 is unlikely to be protonated externally through other titratable amino acids.

Although the major PsbA substitutions are in the vicinity of RC pigments, there are a few notable differences closer to the OEC as well as to Q_B_. The Cl-1 water channel leading to the Mn4 atom of the OEC begins at the D1–PsbO interface and includes several conserved D1 residues (Y73, E65, D59, R334, D61, and N181). Recent crystallographic models and kinetic measurements (38) suggest that the D1-P173M mutation led to a narrower Cl-1 channel in PsbA2 and disappearance of two waters near N181 possibly leading to slow proton egress during the S_1_-S_2_-S_3_ transition in PsbA2-PSII. However, we did not observe any loss of crystal waters within the timescales of our simulations. In conclusion, the most important structural changes in the three variants appear to be in the vicinity of Pheo_D1_, with the presence of a protonated glutamate in the D1-130 position being the most prominent difference.

### Redox Tuning of the Primary Radical Pair.

Our results show that the protein matrix can adjust the redox properties of both pigments involved in primary charge separation, while protein matrix dynamics can also induce redox potential differences. For the primary acceptor, the computed trends in EA (Pheo_D1_^–^/Pheo_D1_) suggest that Pheo_D1_ in A3 have the highest EA, but the trends in PsbA2 is not clearly distinguishable from A1. It has been suggested that Pheo_D1_ has a higher reduction potential in PsbA3 than PsbA1/A2 (30, 38, 39, 75), which is consistent with our results. Moreover, we find that multiple substitutions affect the EA (Pheo_D1_^–^/Pheo_D1_) differently in PsbA3 and A2, despite both having the same D1-130E substitution. Spectro-electrochemical measurements by Sugiura et al. (30) reported a more positive *E*_m_ of Pheo_D1_ in PsbA3-PSII (−505 mV) compared to PsbA1 (−522 mV) (75, 77). This finding was partly attributed to the stronger H-bond between Pheo_D1_–E130 in PsbA3 compared to Pheo_D1_–Q130 in PsbA1. FTIR spectroscopy and DFT analysis also indicated shifts in *E*_m_ (Pheo_D1_^–^/Pheo_D1_) upon modification of a H-bond donor in PsbA1 and A3 (73), but the past computational analysis relied upon implicitly solvated models of Pheo_D1_ without accounting for the native protein environment. The *E*_m_ (Pheo_D1_^–^/Pheo_D1_) of PsbA2-PSII remained unknown until a recent study by Boussac and coworkers reported it to be lower by ~30 mV in PsbA2 (−535 mV) compared to PsbA3. This was estimated by the energy gap between S_2_Q_A_^–^ and P_680_^+^Pheo_D1_^–^ (38) based on thermoluminescence and kinetics of proton release. It is important to note that the exact IE/EA differences cannot be directly compared with reported redox potential differences due to the difficulty in aligning the results of experimental and theoretical methods. For instance, spectroscopic measurements of *E*_m_ (Pheo_D1_^–^/Pheo_D1_) are often performed in dithionite-treated PSII samples, possibly not reflecting the physiological state of the RC, as Q_A_ is reduced to Q_A_^–^ (“closed” RC) (30, 75). The reported values thus assume a consistent electrostatic effect of Q_A_^–^ (c550 bandshift) across all PsbA variants but the shifts induced by Q_A_^–^ may be significant for the absolute differences of *E*_m_ (Pheo_D1_^–^/Pheo_D1_).

Experimental E_m_ values for the (Chl_D1_^+^/Chl_D1_) redox couple are not directly available. The stability of the Chl_D1_^+^Pheo_D1_^–^ state can be inferred however from relative differences in the IE(Chl_D1_^+^/Chl_D1_) and EA(Pheo_D1_^–^/Pheo_D1_) (*SI Appendix*, Fig. S4*C*), and the total free energy change (∆*G*) associated with formation of Chl_D1_^+^ and Pheo_D1_^–^ from Chl_D1_ and Pheo_D1_, respectively. Our results indicate that the Chl_D1_^+^Pheo_D1_^–^ state would be most stable in PsbA3-PSII (∆*G* most negative) followed by PsbA1 and PsbA2. This trend aligns with CT energetics (*SI Appendix*, Fig. S7), showing a correlation between the extent of charge transfer in the excited state and the redox properties of the Chl_D1_–Pheo_D1_ pair. Specifically, it can be expected that formation of the Chl_D1_^+^Pheo_D1_^–^ state would be facilitated in PsbA3-PSII more than in PsbA1 or PsbA2. It is important to emphasize that the employed energy values represent vertical energy differences upon electron attachment or detachment to/from the QM subsystem. However, to accurately determine the redox potentials of the pigments and the free energies of charge-separated states, additional energetic and entropic contributions would need to be considered across a larger protein ensemble from extended MD simulations while also taking into account variations in local protein–pigment interactions. This represents a considerable and largely unmet theoretical and computational challenge, therefore at the moment one-to-one correspondence between calculation and experiment cannot be established. Despite these complexities, the present findings qualitatively align with past experimental studies on the stability of the Chl_D1_–Pheo_D1_ pair (30, 38–40, 83), thus supporting the relative differences across the PsbA variants.

### Spectral Tuning of Chl_D1_–Pheo_D1_ Excited States.

The electrostatic effects of the D1 variants are not only important to the intrinsic absorption properties of the pigments but also toward pushing the red limit of PSII. The specific D1-Q130E substitution exists in most cyanobacterial PsbA variants, especially those acclimated to high-light conditions (PsbA3). Interestingly, the exact Gln/Glu substitution is observed in Far Red Light Photoacclimated (FarLiP) cyanobacteria (86) and some higher plants (27). Consequently, specific variants of core PSII proteins available to different organisms are utilized to adjust photosynthetic efficiency in response to environmental conditions not only by presenting alternate localized electrostatic contributors to critical pigments like in the case of *psbA* variants but also by adopting different conformations within the same variant. The present results demonstrate how the local electrostatic environment and conformational changes in the D1 protein matrix are responsible for enabling access to low-energy CT states in all D1 variants. Importantly, the shifts in Chl_D1_*^δ^*^+^Pheo_D1_*^δ^*^–^ CT states and primary charge separation cannot be explained by a limited number of substitutions but result from global electrostatic optimization.

**Fig. 4.**
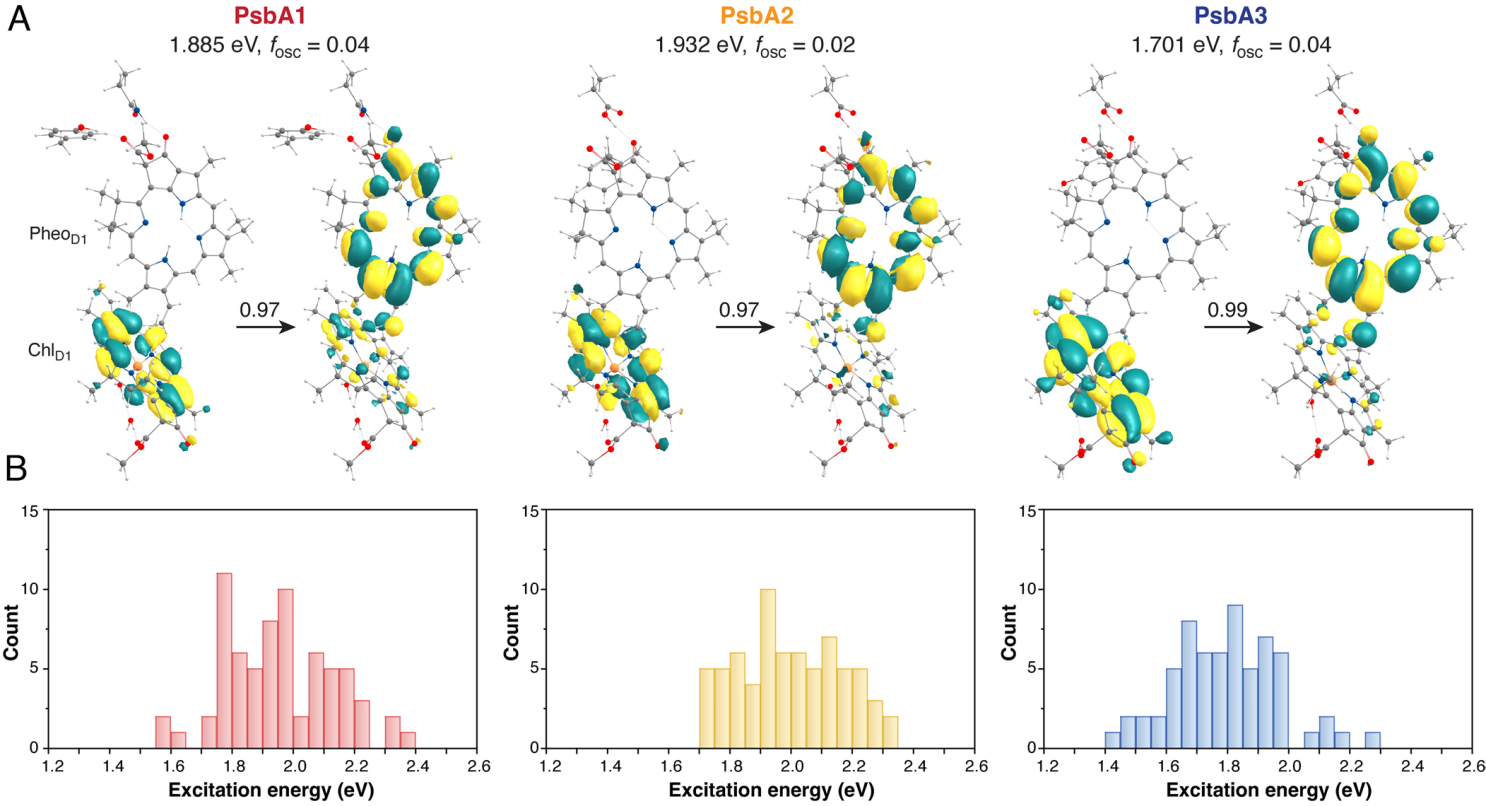
(*A*) Detailed description of the nature and identity of the lowest excited state with significant Chl_D1_*^δ^*^+^Pheo_D1_*^δ^*^–^ CT character (snapshot 1) in terms of donor and acceptor NTOs and relative contributions to a given excitation. (*B*) Relative distribution of the lowest state with dominant Chl_D1_*^δ^*^+^Pheo_D1_*^δ^*^–^ CT character in the PsbA variants. The QM(TD-DFT)/MM excited state calculations are performed on nine different QM/MM geometries distributed among 65 independent protein snapshots obtained from MD simulations in each variant. Detailed results, including vertical excitation energies and oscillator strengths, are provided for each state in *SI Appendix*, Table S7.

Mutagenesis and spectroscopic studies (40, 87) reported that Q_A_ reduction induces a red shift (~3.0 nm) in the Pheo_D1_ Q*_x_* band (C550 bandshift) for PsbA3-PSII (547.3 nm), relative to PsbA1 (544.3 nm). This is attributed to the stronger H-bond to the keto of the Pheo_D1_ from the carboxylate group of D1-130 in PsbA3-PSII than that of PsbA1-PSII. The bandshift for PsbA2-PSII was proposed to be similar to that in PsbA3-PSII assuming that the same residue (D1-130) is responsible for the spectral shift in both proteins (38, 39). Studies on *Synechococcus* PCC7942 and *Synechocystis* PCC6803 reported a 25% increase in the quantum yield of primary CS with the high light D1 isoform (PsbA3) compared to the low light isoform (PsbA1), as well as with the Q130E mutation in PCC6803 (71–73). These findings align with previous studies indicating higher CS yields in WT-PsbA3 than E130Q mutants in *C. reinhardtii* (79, 81). Our results are consistent with the above experimental observations, but we attribute the differences to a combined effect of both redox and spectral tuning of the Chl_D1_–Pheo_D1_ pair by D1 electrostatics. First, we showed that Pheo_D1_ is more easily reduced (high EA) in PsbA3 than PsbA1/A2 (*SI Appendix*, Fig. S4), Second, the protein matrix in PsbA3 shifts Chl_D1_*^δ^*^+^Pheo_D1_*^δ^*^–^ states toward longer wavelengths (Fig. 4*B*). This suggests that the PsbA3 protein tunes the EA of Pheo_D1_ to enhance forward ET and increase CS. These results are physiologically significant because not only PsbA3 exhibit increased phototolerance in cyanobacteria but also its PsbA homolog is most prevalent in eukaryotic PSII (27). While the connection between photoprotection mechanisms and primary CT energetics cannot be directly inferred here, it is hypothesized that unlike PsbA1/A2, the more stable [P_680_^+^Pheo_D1_^–^] in PsbA3 likely favors direct charge recombination over dissipation via the triplet route (74).

**Fig. 5.**
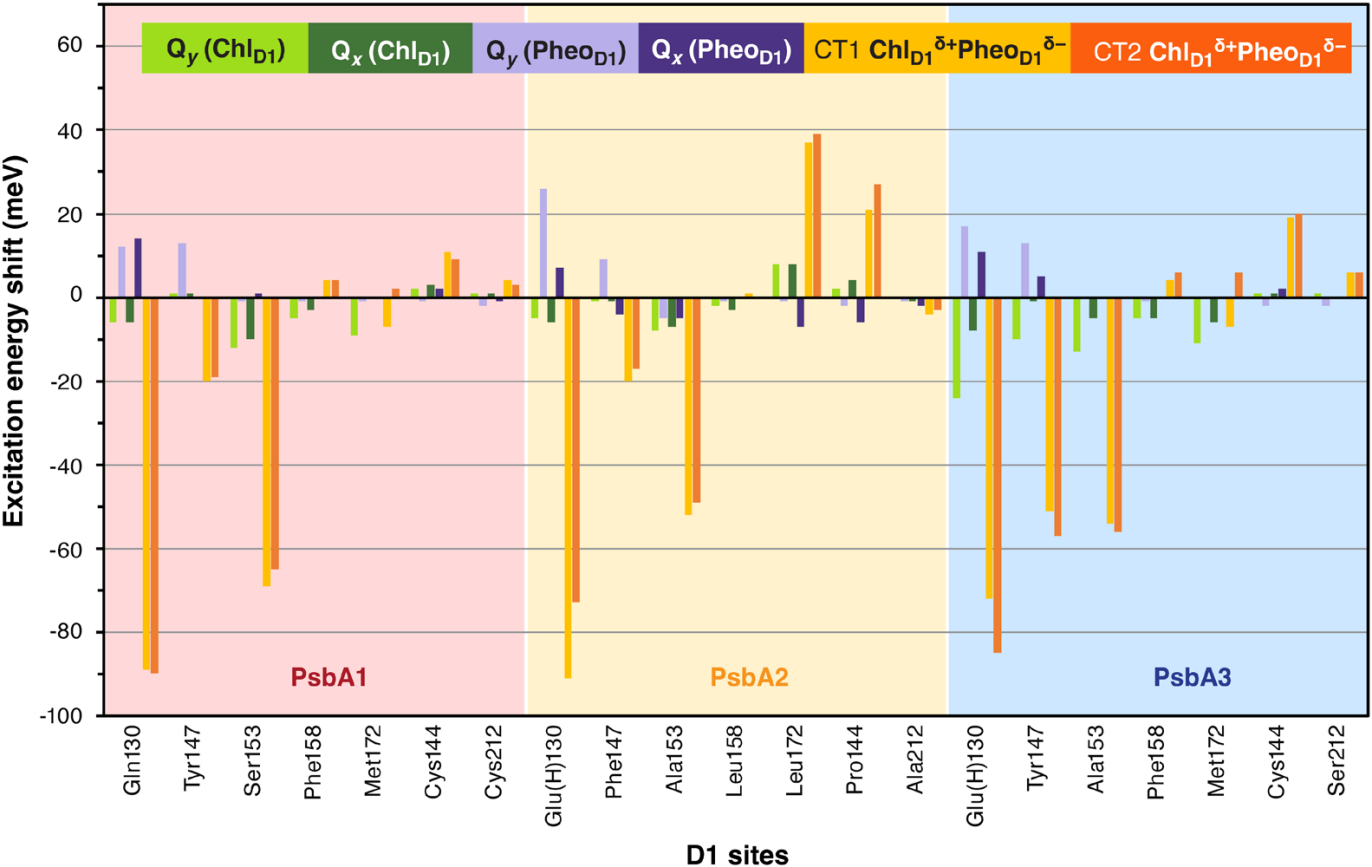
Electrostatic contributions of selected D1 residues within PsbA1, PsbA2, and PsbA3 proteins to the overall electrochromic shifts of the first two local excitations and the two lowest CT states of the Chl_D1_–Pheo_D1_ pair. Negative values indicate a red-shift and positive values indicate a blue-shift. Complete data are provided in *SI Appendix*, Table S8.

In PsbA1/A2, our results do not show major differences in EA (Pheo_D1_^–^/Pheo_D1_) but the IE (Chl_D1_^+^/Chl_D1_) (*SI Appendix*, Fig. S4) and excitation profiles of the Chl_D1_–Pheo_D1_ pair vary. In PsbA2-PSII Chl_D1_ is a poorer donor (higher IE) and Pheo_D1_ is a poorer acceptor (lower EA) (*SI Appendix*, Fig. S4), which blue-shifts the Chl_D1_*^δ^*^+^Pheo_D1_*^δ^*^–^ CT states (Fig. 4*B*). Interestingly, the D1 matrix in PsbA2 is found to inherently disfavor low-energy CT states at longer wavelengths, which could possibly lead to lower yields of primary CS within PsbA2 under normal light conditions compared to PsbA3 and PsbA1. This suggests that primary CS is more constrained in PsbA2 possibly leading to slower ET kinetics and O_2_ evolution. While there are no direct experimental observations on the primary charge separation in PsbA2-PSII, some studies (38, 39) have reported a lower yield of O_2_ production and slow Y_Z_ oxidation linked to specific PsbA2-mimicking substitutions on the donor side. Further spectroscopic and mutagenesis studies are needed to fully elucidate the physiological relevance of these effects. A deeper understanding of the thermodynamics and kinetics of primary charge separation necessitates free energy calculations that account for solvent reorganization, redox coupling, and protein relaxation. The present findings already provide strong evidence that spectral tuning of the primary CT state within the PsbA2 or PsbA3 protein matrix plays a crucial role in modulating the efficiency of charge separation and subsequent electron transfer.

## Conclusion

Atomistic molecular dynamics combined with multiscale QM/MM calculations reveal how PsbA genetic variants modulate property–function relationships in the reaction center pigments of cyanobacterial PSII. Multiscale simulations and structural analysis demonstrate that the protonation state of D1-E130 in the PsbA2 and A3 variants is key for determining the stability of the Pheo_D1_ acceptor, which in turn influences primary charge separation. The hydrogen bond between Pheo_D1_ and D1-130 fine-tunes the optical and redox properties of the Chl_D1_–Pheo_D1_ pair across all three variants. Importantly, the spectral tuning of the Chl_D1_*^δ^*^+^Pheo_D1_*^δ^*^–^CT state is not due to a single substitution but emerges as a cumulative effect of multiple D1 substitutions. Excited state calculations on an ensemble of protein configurations show that PsbA3 red-shifts while PsbA2 blue-shifts the lowest CT state of the RC compared to PsbA1-PSII. Electrostatic effects of specific substitutions were identified, suggesting potential targets for future mutagenesis experiments toward spectral tuning of CT states. Overall, the combined effect of both redox and spectral tuning of the Chl_D1_–Pheo_D1_ pair by D1 matrix electrostatics has functional implications for primary charge separation in each variant. This work illustrates core atomistic principles of how protein electrostatics modulate excited state properties, laying a foundation toward engineered optimization of photosynthetic systems.

## Materials and Methods

Models of each PSII-PsbA variant (PDB IDs: 3WU2, 7YQ2, and 7YQ7) were embedded in a POPC bilayer-water-ion simulation box with 150 mM NaCl buffer, and simulated using atomistic MD with the AMBERff14SB force field (88), and in-house parameters for nonstandard residues (89). The systems were equilibrated and simulated using all-atom MD for 300 ns with a 2 fs time-step at 300 K temperature and 1 atm pressure, using *pmemd.cuda* in Amber20. (90) Binding free energies were calculated using MM-PBSA (54), and trajectories were analyzed using VMD and CPPTRAJ. QM/MM geometry optimizations and redox potential calculations for the RC pigments were performed using DFT/MM, while excited states were computed via long-range corrected TD-DFT with QM/MM on an ensemble of MD snapshots, employing the multiscale module of ORCA 5.0 (91). MD-PMM free energy calculations were performed using the open-source PyMM code (92). Further details of the simulations are provided in *SI Appendix*.

## Supplementary Material

Appendix 01 (PDF)

## Data Availability

Computational results data have been deposited in Edmond, the Open Research Data Repository of the Max Planck Society (https://doi.org/10.17617/3.D57FWE) (93).
