# Supplementary material for: Microscopic basis of reaction center modulation in PsbA variants of photosystem II
Source: Proc Natl Acad Sci U S A. 2025 May 12;122(20):e2417963122. doi: 10.1073/pnas.2417963122 (PMC12107152; doi:10.1073/pnas.2417963122)
Supplement: Supplementary file 1 — Appendix 01 (PDF) [file pnas.2417963122.sapp.pdf]

PNAS

Supporting Information for

Microscopic Basis of Reaction Center Modulation in PsbA Variants of Photosystem II

Sinjini Bhattacharjee,[a] Igor Gordiy,[a] Abhishek Sirohiwal,[b] Dimitrios A. Pantazis*[a]

[a] Max-Planck-Institut für Kohlenforschung, Kaiser-Wilhelm-Platz 1, 45470 Mülheim an der Ruhr, Germany.

[b] Department of Inorganic and Physical Chemistry, Division of Chemical Sciences, Indian Institute of Science, Bangalore 560012, India.

* Corresponding author: Dimitrios A. Pantazis.

Email: dimitrios.pantazis@kofo.mpg.de

This PDF file includes:

Supporting text
Figures S1 to S16
Tables S1 to S8
SI References

Supporting text

Preparation of MM models

Separate MM models for each PsbA variant were constructed to evaluate the effect of substitutions in each D1 copy. The initial structure of the PSII monomer is based on the high-resolution crystal structure of *T. vulcanus* (PDB ID: 3WU2) (1), originally containing the PsbA1 variant. For the PsbA2 and A3 variants, the D1 protein (Chain A of 3WU2) was replaced with the corresponding sequences of PsbA2- and PsbA3-PSII based on the recent crystal structures (PDB ID: 7YQ2 and 7YQ7) (2)**.** We note that the PsbA1 sequences of *T. vulcanus* and *T. elongatus* are identical with the exception of a single difference at D1-286 (3). Two protonation states for the mutation at D1-130 were considered for PsbA2 and PsbA3 variants, namely (a) D1-Glu for the deprotonated glutamate, and (b) D1-Glu(H) for glutamic acid. In each case, the complete protein unit was embedded in a 1-palmitoyl-2-oleoyl-*sn*-glycero-3-phosphocholine (POPC) lipid bilayer using Packmol-Memgen (4, 5) and solvated with TIP3P waters (6) (**Fig. S1**). The number of lipids and water molecules were kept same for each setup. All models were neutralized with appropriate number of counterions and a 0.15 M physiological Na^+Cl^- salt concentration. For standard protein residues, waters, and the lipid bilayer we used parameters from the AMBERff14SB (7-12), TIP3P, and Lipid17 (13) force fields, respectively. The partial charges and force field parameters for the organic cofactors (plastoquinones, carotenoids, structural lipids) were obtained using GAFF2 (7, 9), parameters for the OEC were taken from earlier studies (14, 15), while those of the remaining cofactors (non-heme Fe, chlorophylls, pheophytins and hemes) (14, 16, 17) were obtained from the literature (18-20).

Molecular Dynamics (MD) simulations

A step-by-step minimization protocol was employed to remove energetically unfavorable geometric clashes in the system. In the equilibration phase, the systems are heated from 10 to 100 K in a succession of 5 ps in the NVT ensemble and further from 100 K to 303 K in the NPT ensemble for 125 ps. The temperature during this step is maintained using Langevin dynamics (21) with a collision frequency of 5 ps^{-1}. During the equilibration phase, the C_α atoms of amino acids were restrained with a force constant of 20 kcal mol^{-1} $Å^{-2}$. Subsequently, the restraints on the C_α atoms of amino acids were systematically decreased (2 kcal mol^{-1} $Å^{-2}$ / 500 ps). The systems were further equilibrated for 65 ns to properly equilibrate the lipid bilayer. Thereafter, a series of production runs were initiated for 60 ns for each system (300 ns in total) without restraints and the temperature and pressure set at 303 K and 1 atm, respectively. During the entire procedure, the temperature was controlled using Langevin dynamics with a collision frequency of 1 ps^{-1} and the system pressure was controlled using the Berendsen

barostat (22) with anisotropic pressure scaling with a relaxation time of 2 ps. The SHAKE algorithm (23) was used to constrain the bonds involving hydrogens. The MD time step was 2 fs and frames were saved every 20 ps. The electrostatic interactions were treated using the Particle Mesh Ewald (PME) (24-27) approach with a 10 Å cut-off. AMBER20 (28) was used to perform the energy minimizations and equilibration dynamics. The production MD simulations were performed in the GPU version of the pmemd module (*pmemd.cuda*) and trajectory analysis was performed using *Cpptraj* in AmberTools (28-30), VMD (31) and PyMol (32).

Binding Free Energy Calculations

In order to compare the thermodynamic stability of $Pheo_{D1}$ in each D1 isoform, binding free energies were calculated using the MM-PBSA (33) module in AmberTools. $Pheo_{D1}$ is considered as the ligand and the D1/D2 protein couple as the receptor. MM-PBSA combines molecular mechanics, implicit Generalized Born (GB) / Poisson–Boltzmann (PB) solvation schemes and solvent accessibility surface area calculations to estimate the binding free energies of a protein-ligand complex. A single trajectory approach is employed, where only the complex form is propagated, eliminating the need for separate molecular dynamics (MD) simulations for the ligand and protein. Solvation free energies are computed using both the GB and PB solvation schemes. Binding energy calculations for each MM system were carried out on 300 equidistant snapshots extracted from the initial 60 ns of production MD. The calculated binding energies were subsequently averaged over this ensemble. The solute dielectric constant is set to 2.0 in all cases. Our primary goal was only to compare the relative stabilities of $Pheo_{D1}$ in each PsbA matrix, so the entropic contribution to the binding energy is not computed due to its high computational cost. The calculations are conducted for all the D1 isoforms. The binding free-energies were decomposed into per-residue contributions to assess the influence of each residue towards the binding stability of $Pheo_{D1}$ in each PSII variant. All calculations were performed using the parallelized *MMPBSA.py.MPI* module of the Amber20 package (28).

QM/MM Geometry optimizations

We chose 3x9 independent MD snapshots for QM/MM optimizations. The first snapshot resembles the “crystal like” structure from the initial equilibration, while the remaining 8 were taken from the production MD of each PsbA1/A2/A3- PSII protein. For the QM/MM calculations the complete PSII monomer and all waters around the protein were retained (7 Å bulk-region, 8000 water molecules including internal cavity waters). Appropriate number of Na^+ ions were included to maintain overall neutrality of each system. QM/MM calculations were performed using an electrostatic embedding scheme. The hydrogen link atom approach was employed to cut through C–C covalent bonds; the charge-shift method was used to avoid over-

polarization of QM atoms by the MM charges. The chlorin macrocycles and the axially coordinated ligands to the Mg^{2+} ions were treated at the QM level. For Chl_{D1}, the water molecule that is hydrogen-bonded to the axially ligated water and ester group attached to the 13^2-carbon position on ring E, and all H-bonded sidechains in the vicinity of the Chl_{D1}–$Pheo_{D1}$ pair, were also included in the QM region. The phytyl chains were included up to C-17 (truncated as methyl) and the remaining chain was kept in the MM region. The complete system was further subdivided into two parts: active and static. The active region consisted of all atoms within the QM region as well as atoms within the MM region that are free to move during optimization, whereas the remaining MM atoms were fixed and only contributed to the electrostatics. The Chl_{D1}–$Pheo_{D1}$ and P_{D1}–P_{D2} pairs were optimized in their ground state (S = 0) geometry, for all D1 variants. Complete amino acid residues and waters within 10 Å from the center of each chlorin ring were considered in the active region. The Perdew-Burke-Ernzerhof (PBE) functional (34) was used to optimize the QM regions using the def2-TZVP basis set (35), along with D3(BJ) dispersion corrections (36, 37). Dense DFT integration grids (DefGrid2 in ORCA) were employed; the resolution of identity approximation (RI) (38) was used with the corresponding auxiliary basis sets (def2/J) (39).

Excited State Calculations

The vertical excitation energies (8 roots) were computed on the pair-optimized ground state geometries using QM/MM and full TD-DFT i.e., without the Tamm–Dancoff approximation (TDA). All calculations are performed using the range-separated ωB97X-D3(BJ) functional (modified version of ωB97X-V (40) with D3BJ correction) and the def2-TZVP basis sets, previously benchmarked for similar systems (41). The RIJCOSX approximation (42) and the def2/J auxiliary basis sets (39) were used throughout. VeryTightSCF convergence criteria were applied, along with dense integration grids. To explicitly compare the effect of D1 substitutions and have a meaningful sampling of the protein conformation, further excited state calculations were performed on 65 snapshots obtained from unbiased MD production runs of the PSII-membrane complex, i.e., without any backbone restraints. The electrostatic effects of the protein environment on excited states were included through explicit MM point charges.

The nature of the excited states was determined using Natural Transition Orbitals (NTOs) (43) using the *orca_plot* module. NTOs offer a clearer and more intuitive representation of orbitals involved in hole-particle excitations, particularly in highly delocalized chromophores or multi-chromophoric systems like in this work. These systems often feature excitonic states composed of linear combinations of localized excitations, making it challenging to describe excitations using canonical molecular orbitals. NTOs are derived by applying separate unitary transformations to the occupied and virtual orbitals, providing a localized view of the transition density matrix for each excited state. Unlike attachment/detachment density methods, NTOs

retain phase information, which is valuable for analyzing the diabatic nature of excited states in complex systems. Even when multiple significant NTO amplitudes exist, it still remains the most accurate representation of the particle-hole picture of an excited state.

All QM/MM calculations were performed using the electrostatic embedding scheme as implemented in ORCA 5.0 (44, 45). It is important to note that the choice of embedding scheme is crucial for excited state QM/MM calculations and it is increasingly recognized that fixed charge models (e.g., CHARMM and AMBER) may over-stabilize charge-transfer (CT) states. Future work will explore polarizable embedding approaches to further refine the description of electrostatic interactions, particularly in the context of CT states in the PSII-RC. Nevertheless, we expect that the picture presented here will not be qualitatively modified.

Perturbed Matrix Method (PMM) Calculations

In the Perturbed Matrix Method (PMM) calculations (46-48), the MM system was divided into two parts: a Quantum Center (QC) which is Chl_{D1} or $Pheo_{D1}$, and the environment, which is explicitly treated as a semiclassical perturbation acting on the QC. PMM relies on a long-timescale MD simulation where the entire system is described using a consistent Hamiltonian, i.e., the same force field. Quantum properties of the QC are then recalculated a posteriori for each MD frame using a perturbed Hamiltonian. At each MD frame, a set of eigenvectors and eigenvalues are produced representing the perturbed eigenstates and energies of the QC at a given geometry. In this study, the Gibbs free energy difference is obtained by the equation:

$$\Delta G = -k_B T \ln\langle e^{-\beta\Delta U ox\rightarrow red}\rangle_{ox} = k_B T \ln\langle e^{-\beta\Delta U red\rightarrow ox}\rangle_{red}$$

where k_B is the Boltzmann constant, $U_{ox\rightarrow red}$ and $U_{red\rightarrow ox}$ are the differences between the perturbed energy variation of the whole system upon reduction or oxidation of the cofactor. The energy differences are calculated at each frame of the MD simulation, and the average is evaluated either in the “reduced” or the “oxidized” ensemble, i.e., with the cofactor in either its reduced or oxidized state, respectively, as indicated by the angle brackets subscript. In the above equation, $U_{ox\rightarrow red}$ is estimated through $\Delta\epsilon_{ox\rightarrow red}$, i.e., the energy variation of the whole system is approximated with the difference between the perturbed energy of the QC (the above mentioned eigenenergy) in the reduced and oxidized condition. For simplicity, we used a single MD ensemble (3000 snapshots) for each PsbA variant, that is, with Chl_{D1} and $Pheo_{D1}$ in their neutral states. The unperturbed (gas-phase) ground state energies and dipole moments for Chl_{D1}, Chl_{D1}^{+}, $Pheo_{D1}$ and $Pheo_{D1}^{-}$ were obtained from QM/MM optimized geometries of the individual pigments using the same QM method described above for excited states. All PMM calculations were performed using the modified *pymm* and *free_en* modules of the open-source PyMM code (46).

Supplementary Figures

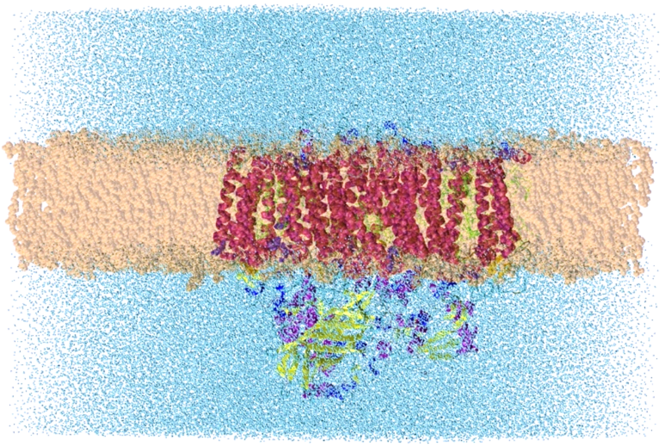

Fig. S1. Side view of the all-atom model of the PSII monomer used in the present study, embedded in the POPC lipid bilayer of dimension 176 × 176 Å^2; salt ions are not shown for clarity.

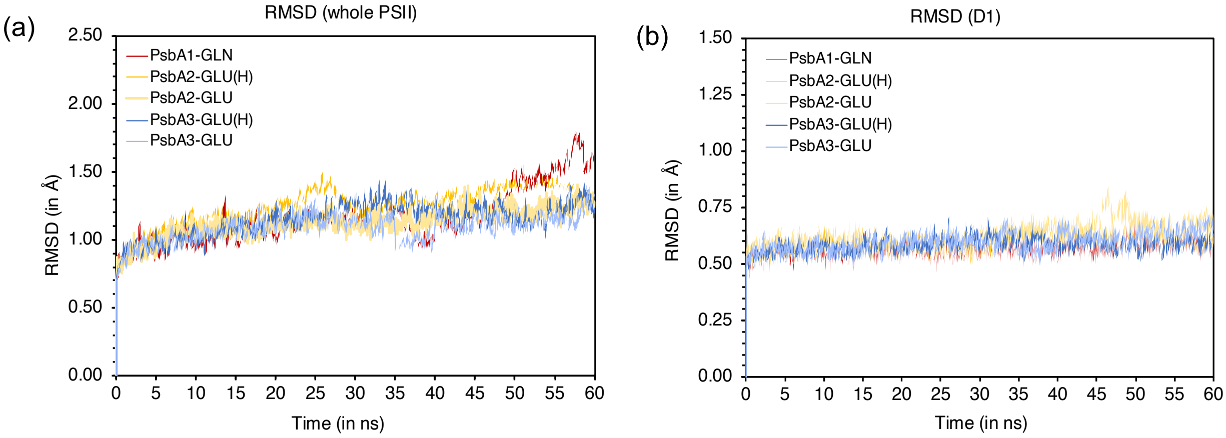

Fig. S2. (a) RMSD (in Å) of the whole PSII protein and (b) RMSD of the PsbA (D1) protein only, for the PsbA1–A2–A3 variants with both protonation states for D1–E130 (values along 60 ns of production MD). Disordered regions of the protein are not considered in the RMSD calculations. The analysis is performed using the Cpptraj module of Amber20.

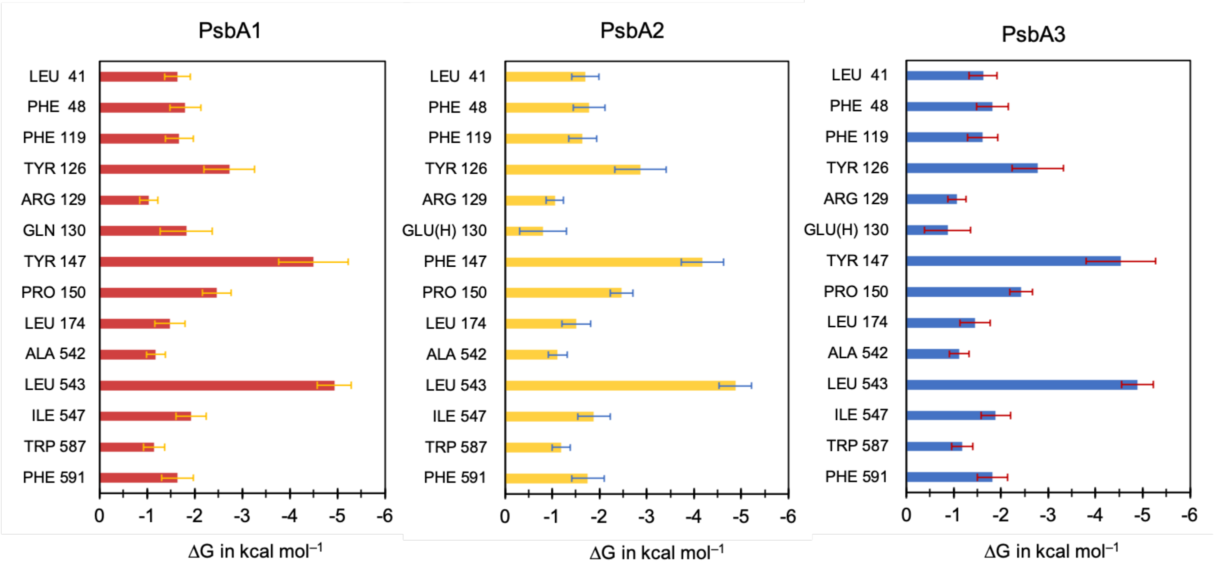

Fig. S3. The total contribution of selected residues to the $Pheo_{D1}$ binding energy calculated with the MM-PBSA approach, for PsbA1–A2-A3. Residues 1–344 belong to the PsbA (D1) protein, while the rest belong to PsbD (D2). The total binding energy is averaged over 300 snapshots across 60 ns of free MD simulations.

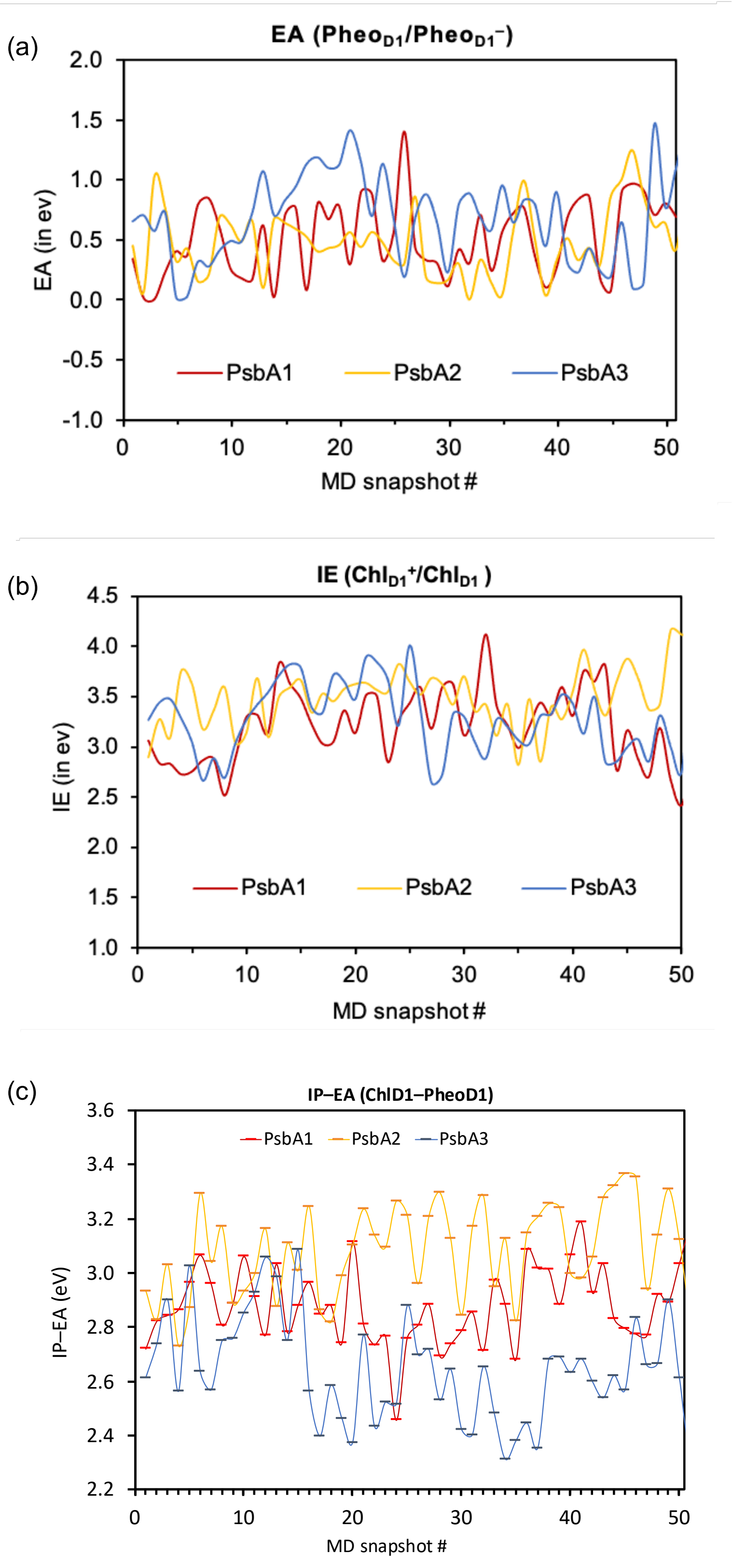

Fig. S4. Vertical (a) Electron affinities (EA) for Pheo$_{D1}^{-}$/Pheo$_{D1}$ and (b) Ionization energies for Chl$_{D1}^{+}$/Chl$_{D1}$ in PsbA1–A2–A3 variants; (c) Difference between vertical IEs and EAs for the Chl$_{D1}$–Pheo$_{D1}$ pair. The QM(DFT)/MM single-point calculations are performed on 50 independent protein snapshots obtained from MD simulations.

Chl_{D1}–$Pheo_{D1}$ (PsbA1)

S1 (1.806 eV), f_{osc} = 0.27 **S2 (1.885 eV), f_{osc} = 0.04** **S3 (2.048 eV), f_{osc} = 0.17**

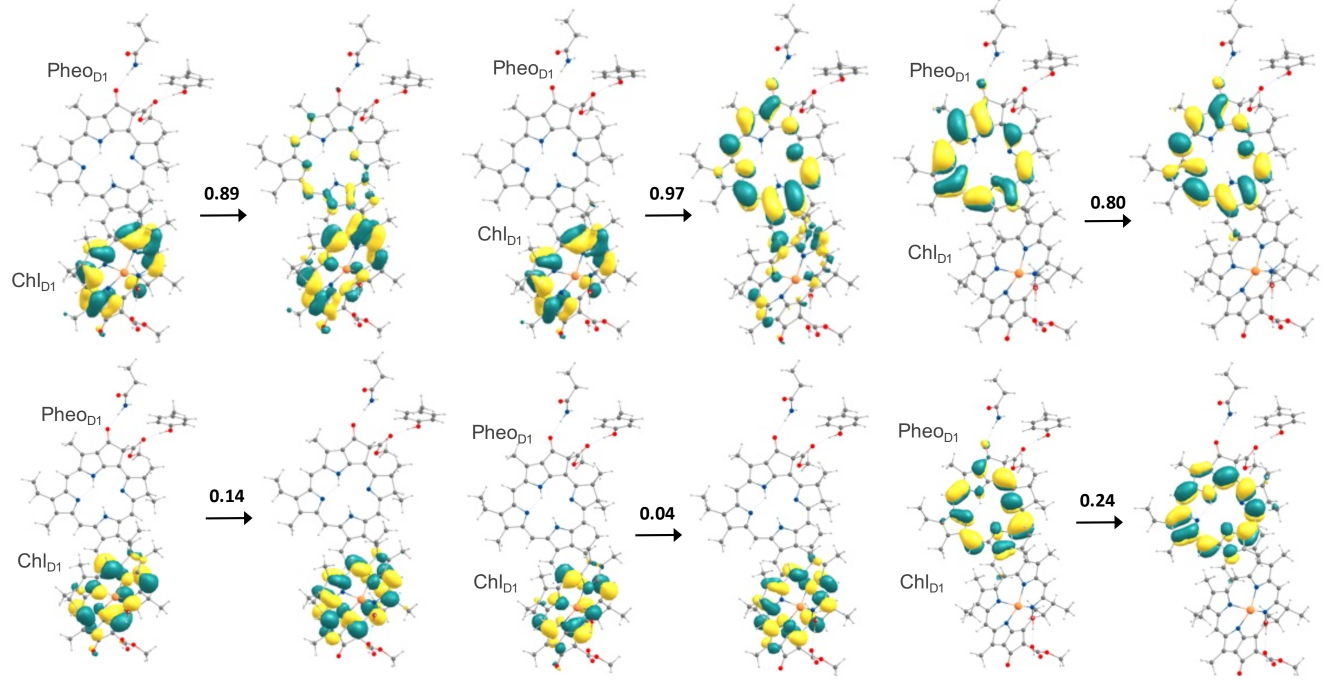

Chl_{D1}–$Pheo_{D1}$ (PsbA2)

S1 (1.816 eV), f_{osc} = 0.28 **S2 (1.932 eV), f_{osc} = 0.02** **S3 (2.029 eV), f_{osc} = 0.17**

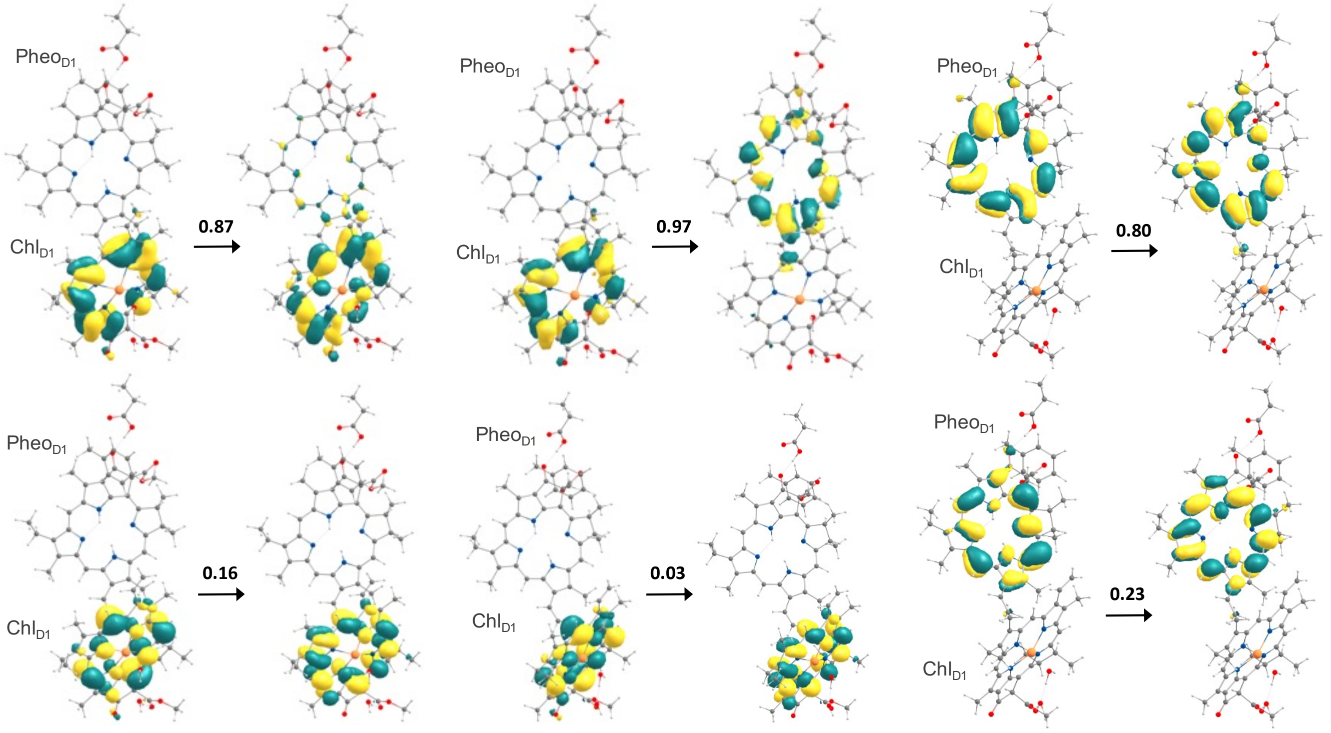

Chl_{D1}–$Pheo_{D1}$ (PsbA3)

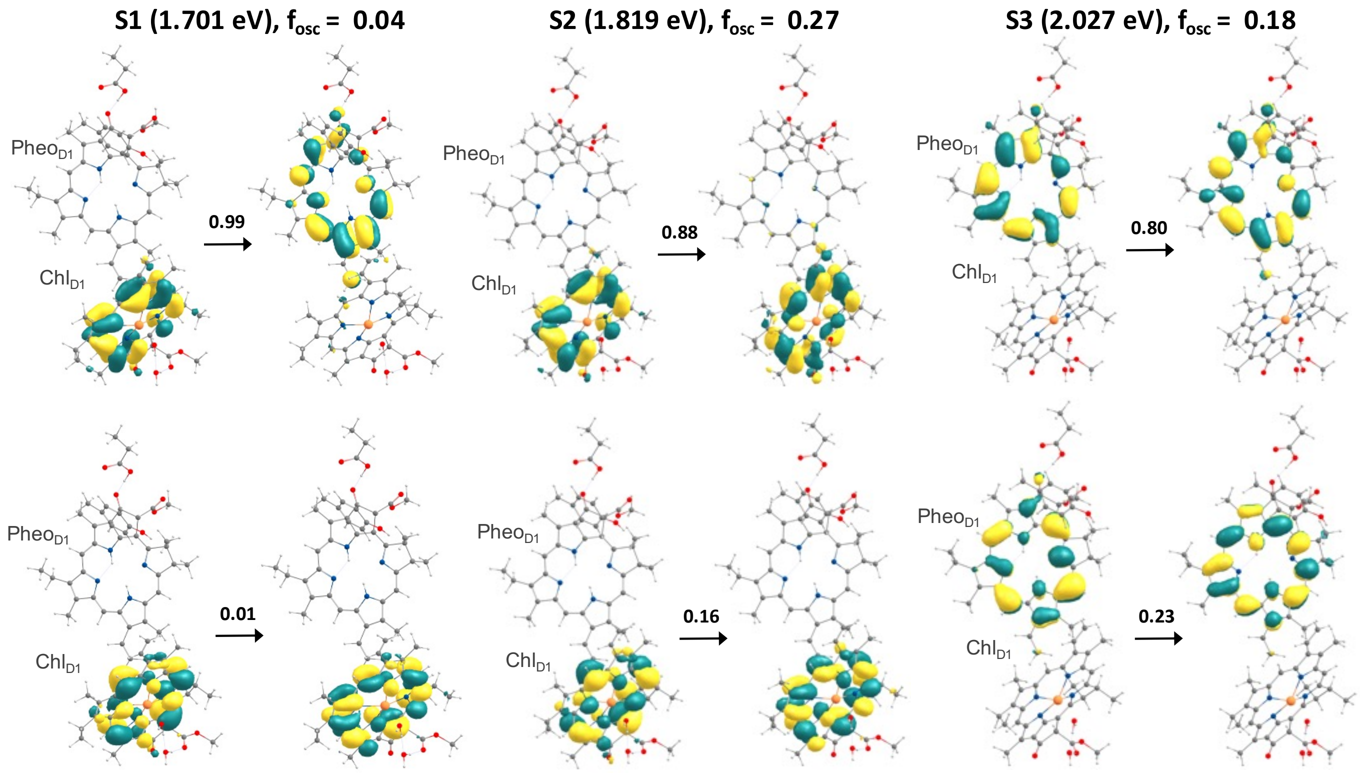

Fig. S5. Identity and nature of the three lowest excited states (S1–S3) for **Chl_{D1}–$Pheo_{D1}$** in terms of Natural Transition Orbitals (NTOs) and relative contributions to a given excitation. Vertical excitation energies (in eV) and oscillator strengths (f_{osc}) are provided for each state depicted (from ωB97X-D3(BJ) TD-DFT calculations), comparing the results in each PsbA-PSII variant.

P_{D1}–P_{D2} (PsbA1)

S1 (1.864 eV), f_{osc} = 0.40

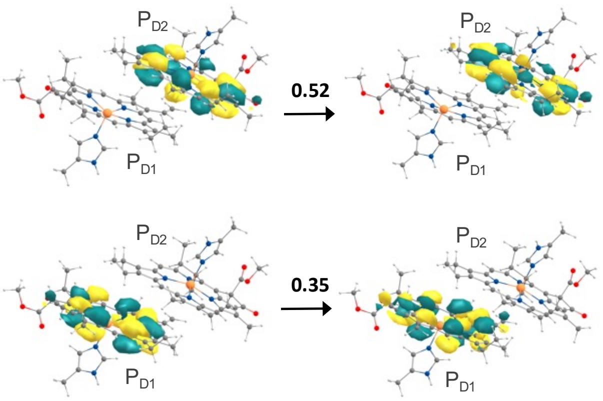

S2 (1.884 eV), f_{osc} = 0.07

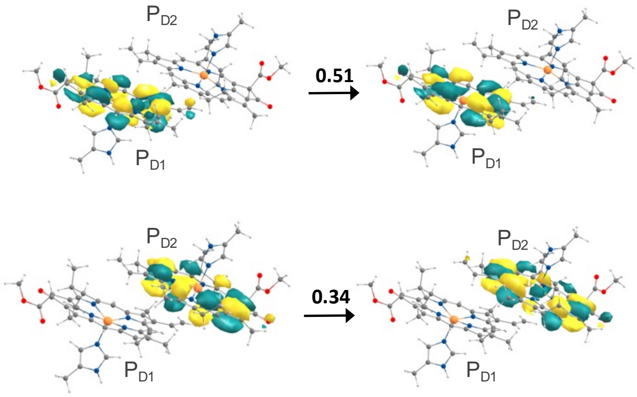

S5 (2.911 eV), f_{osc} = 0.04

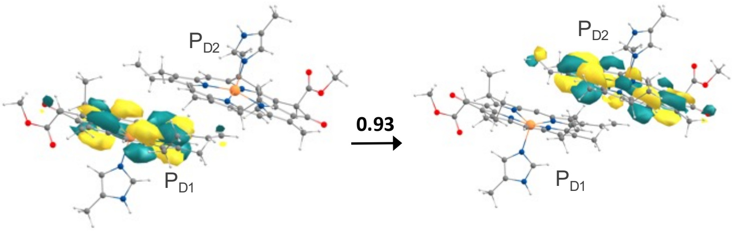

P_{D1}–P_{D2} (PsbA2)

S1 (1.859 eV), f_{osc} = 0.36

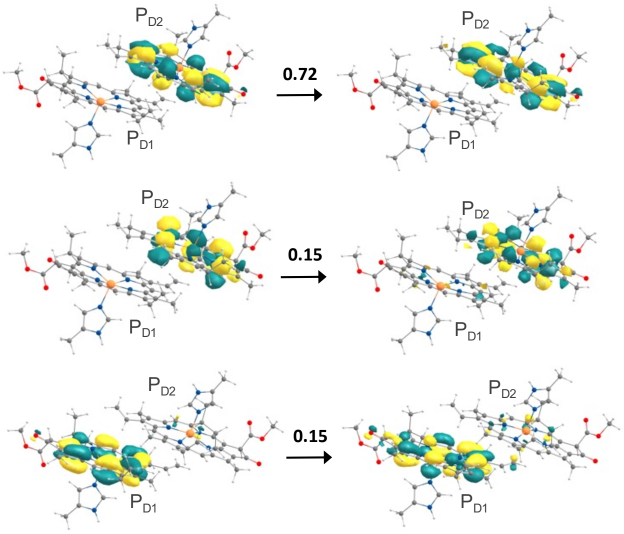

S2 (1.891 eV), f_{osc} = 0.13

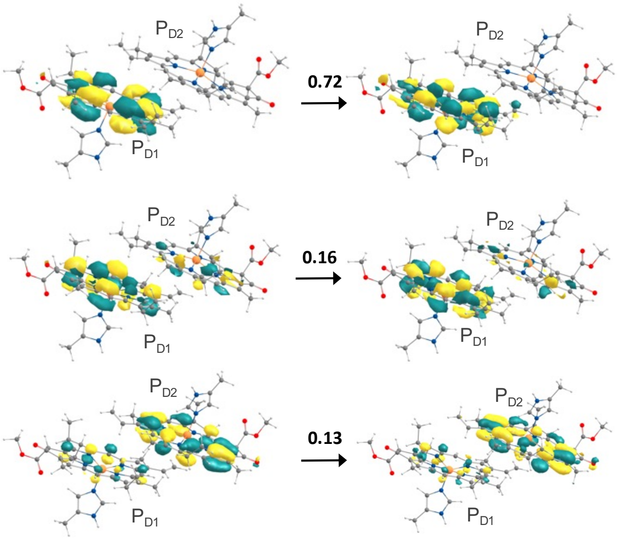

S5 (3.031 eV), f_{osc} = 0.11

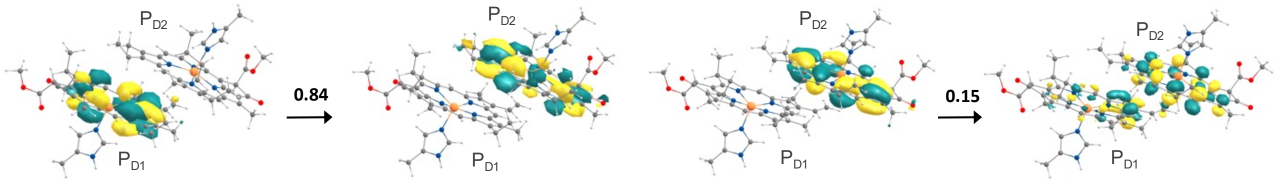

P_{D1}–P_{D2} (PsbA3)

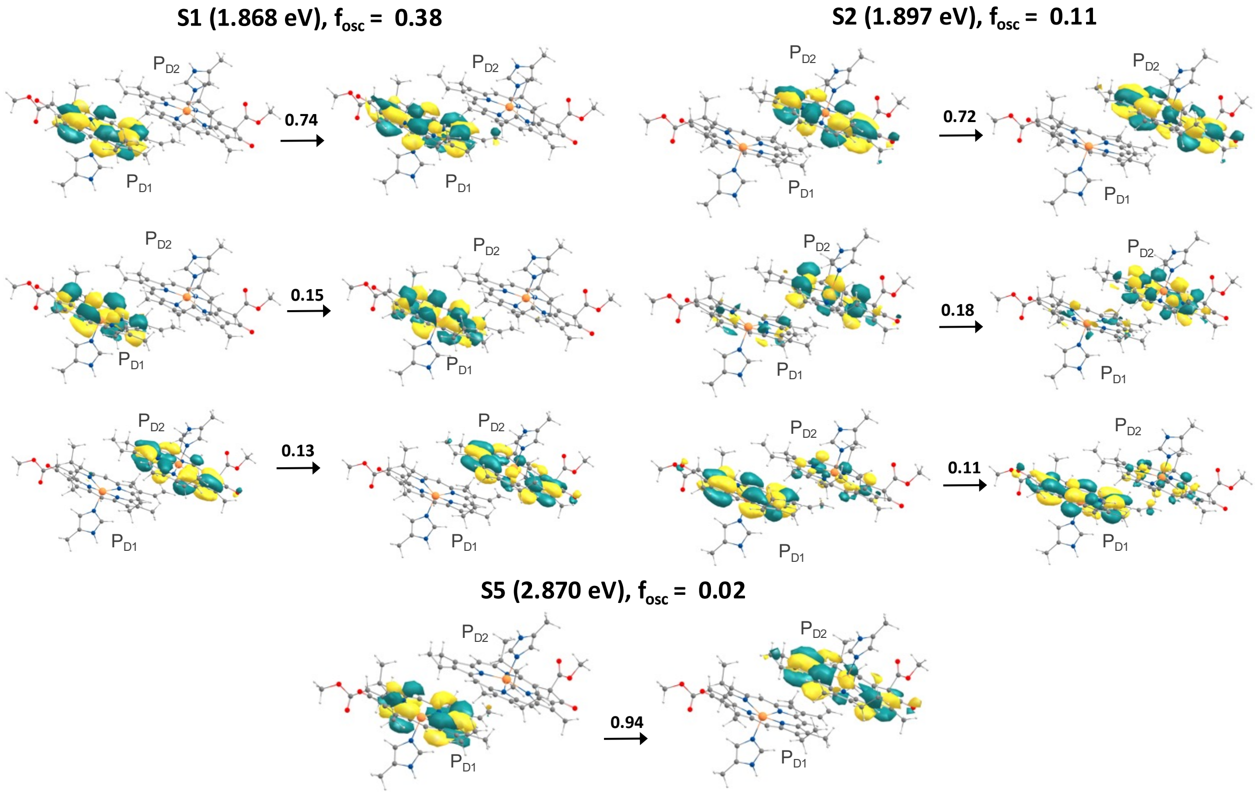

Fig. S6. Identity and nature of selected excited states for the **P_{D1}–P_{D2}** pair in terms of NTOs and relative contributions to a given excitation. Vertical excitation energies (in eV) and oscillator strengths (f_{osc}) are provided for each state depicted (from ωB97X-D3(BJ) TD-DFT calculations), comparing the results in each PsbA-PSII variant.

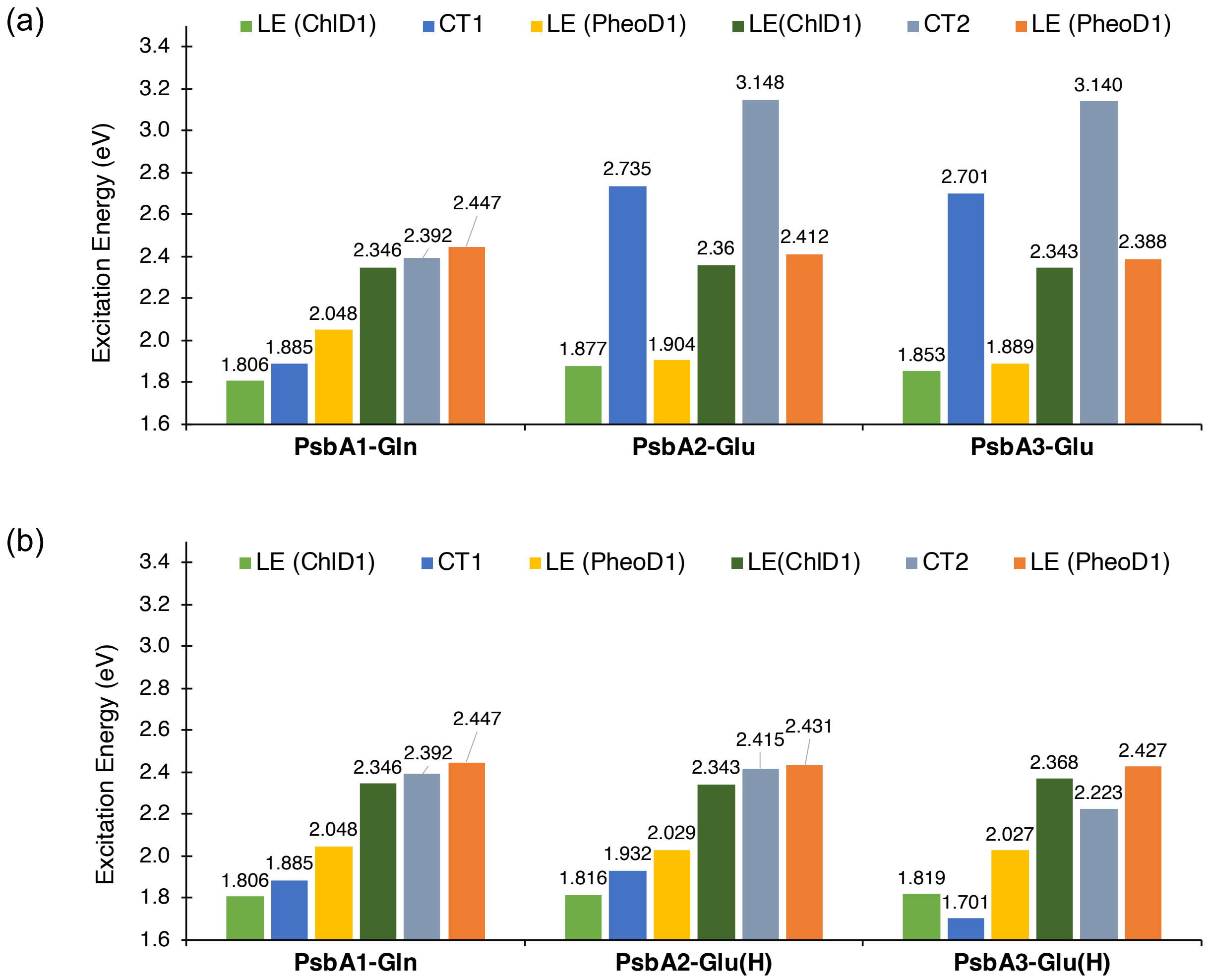

Fig. S7. Comparison of excited state energies on the “crystal-like” conformation (snapshot 1) with D1-Gln130 for PsbA1 and (a) D1-Glu130 (uncharged, protonated) or (b) D1-Glu(H)130 (charged, deprotonated) for the PsbA2 and PsbA3 PSII variants. Chl_{D1} remains the red-most pigment of the RC in all cases. The corresponding NTO compositions and oscillator strengths are described in **Table S4**; the labels depict the nature of each excited state based on the highest NTO coefficient.

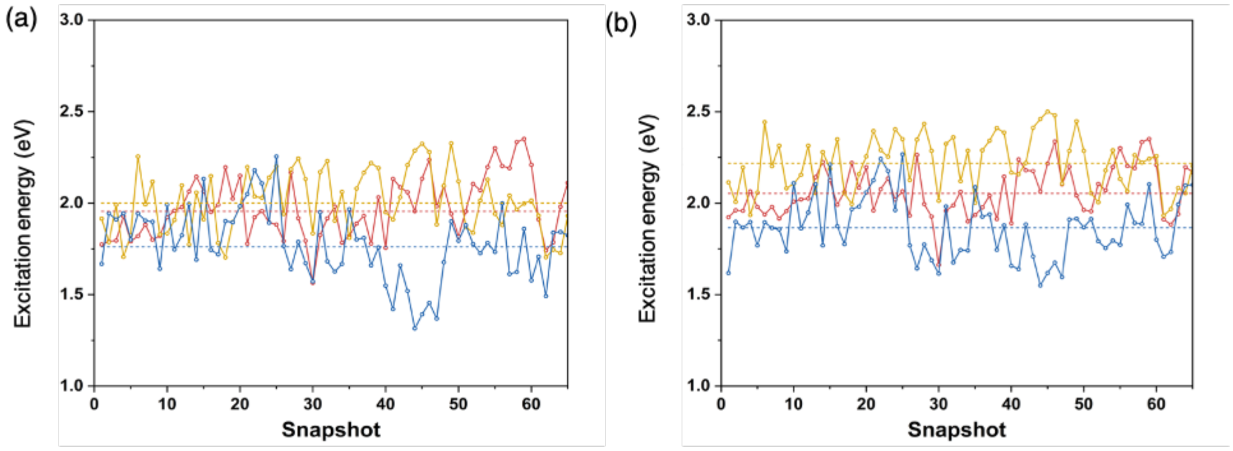

Fig. S8. Excitation energies of the lowest state with dominant $Chl_{D1}^{\delta+}Pheo_{D1}^{\delta-}$ CT character in PsbA1 (red), A2 (yellow) and A3 (blue) variants. The QM(TD-DFT)/MM excited state calculations are performed on 65 independent protein snapshots obtained from MD simulations with (a) 9 independent QM/MM geometries, and (b) a single QM/MM geometry for each variant.

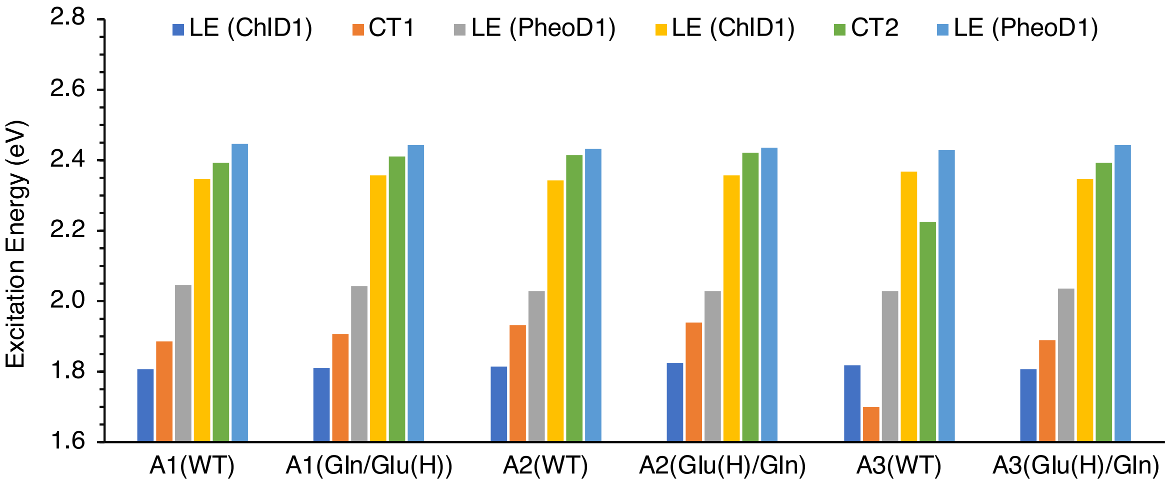

Fig. S9. Comparison of excited state energies on the "crystal-like" conformation for D1-130 (Gln/Glu(H)) variants of PsbA1 and Glu(H)/Gln variants in PsbA2 and PsbA3-PSII, with respect to the wild-type (WT) D1 isoforms in each variant.

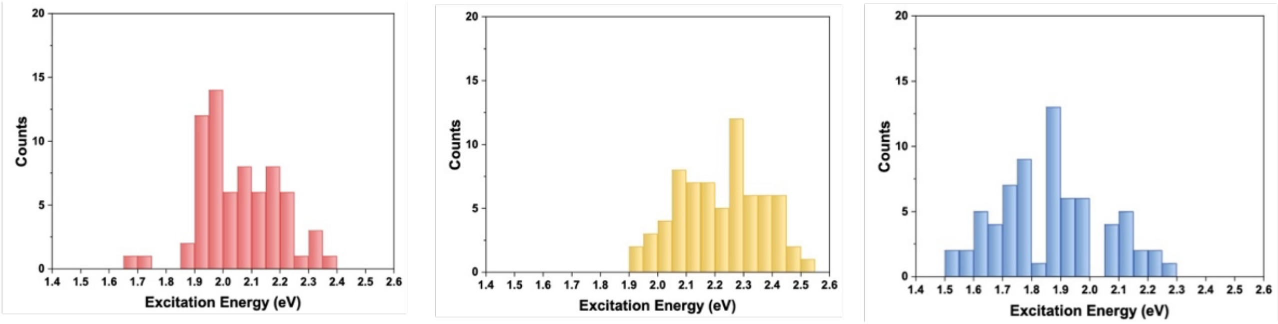

Fig. S10. Relative distribution of the lowest state with dominant $Chl_{D1}^{\delta+}Pheo_{D1}^{\delta-}$ CT character in PsbA1 (red), A2 (yellow) and A3 (blue) variants. The QM(TD-DFT)/MM excited state calculations are performed on a single QM/MM geometry distributed among 65 independent protein snapshots obtained from MD simulations in each case.

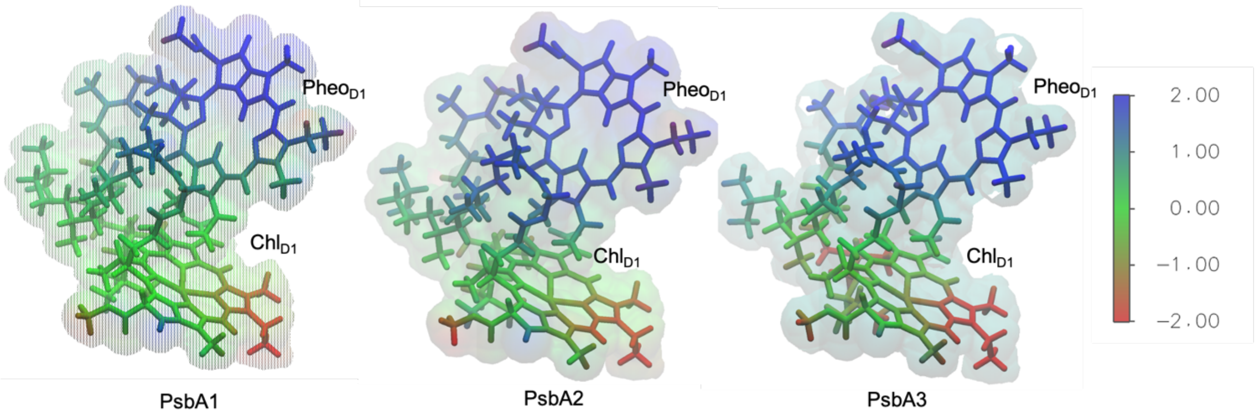

Fig. S11. Electrostatic potential (ESP) experienced by the RC pigments (in kT/e) inside the PSII protein in each variant. The calculations were performed using the APBS (Adaptive Poisson–Boltzmann Solver) in VMD on the crystal-like configuration of the protein in each case.

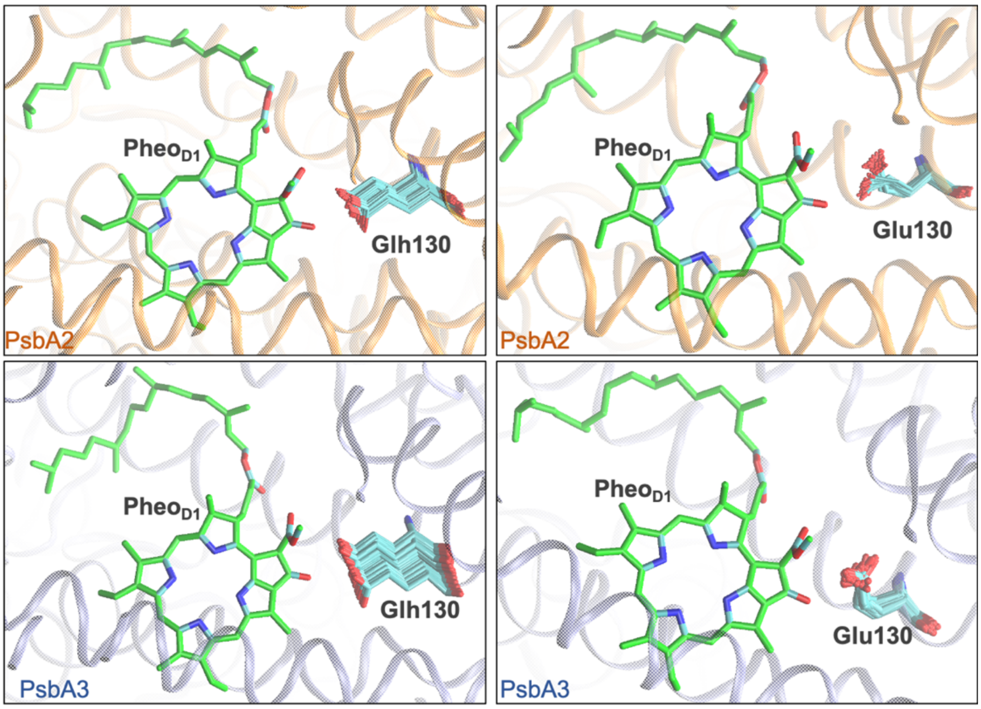

Fig. S12. The effect of protonation of D1-E130 on the orientation of the Glu/Glu(H) side chain during the production molecular dynamics simulations of PsbA2 and PsbA3-PSII. The conformations are averaged over 300 frames across 60 ns MD trajectories for each setup.

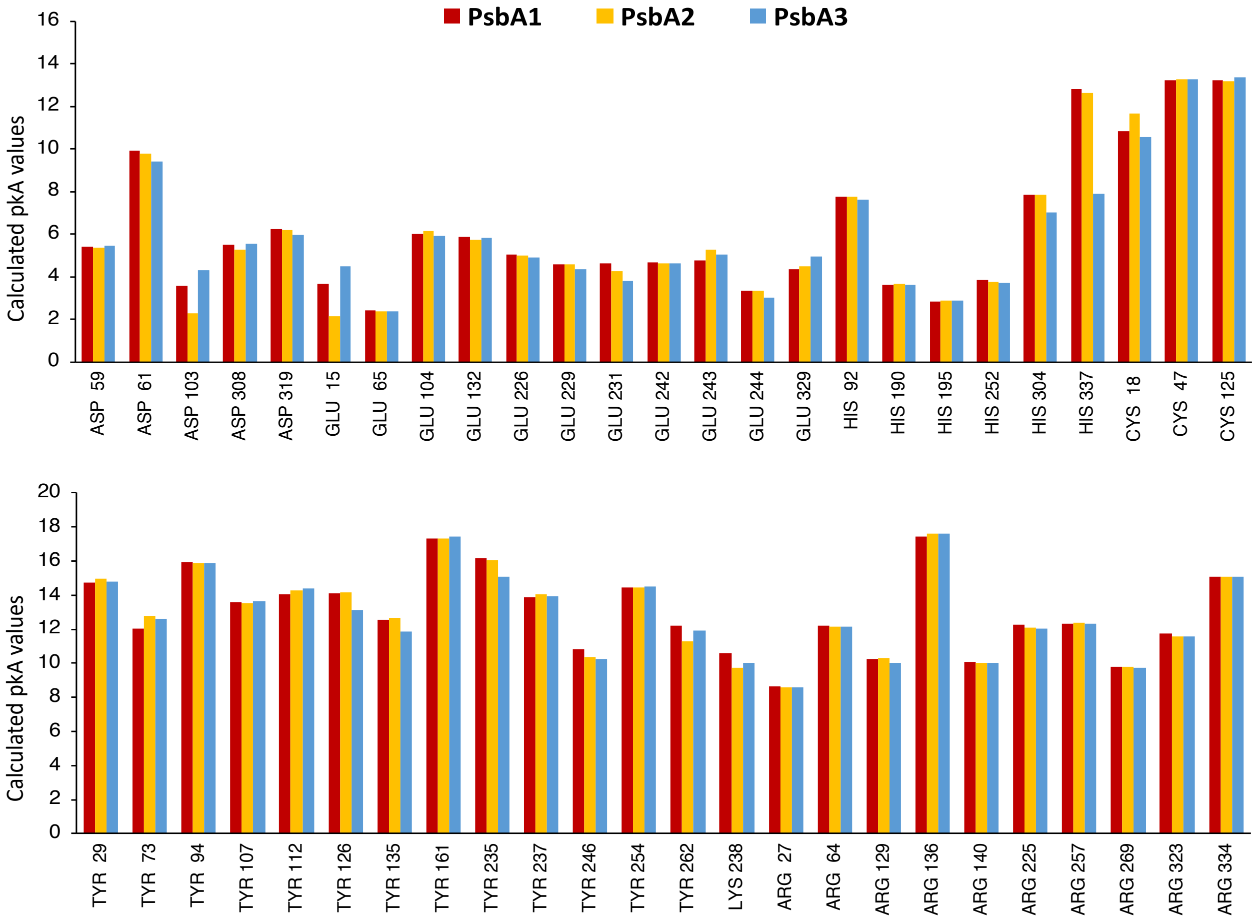

Fig. S13. Estimated pKa values of titratable residues in the PsbA variants, computed using the APBS-PDB2PQR software suite (49).

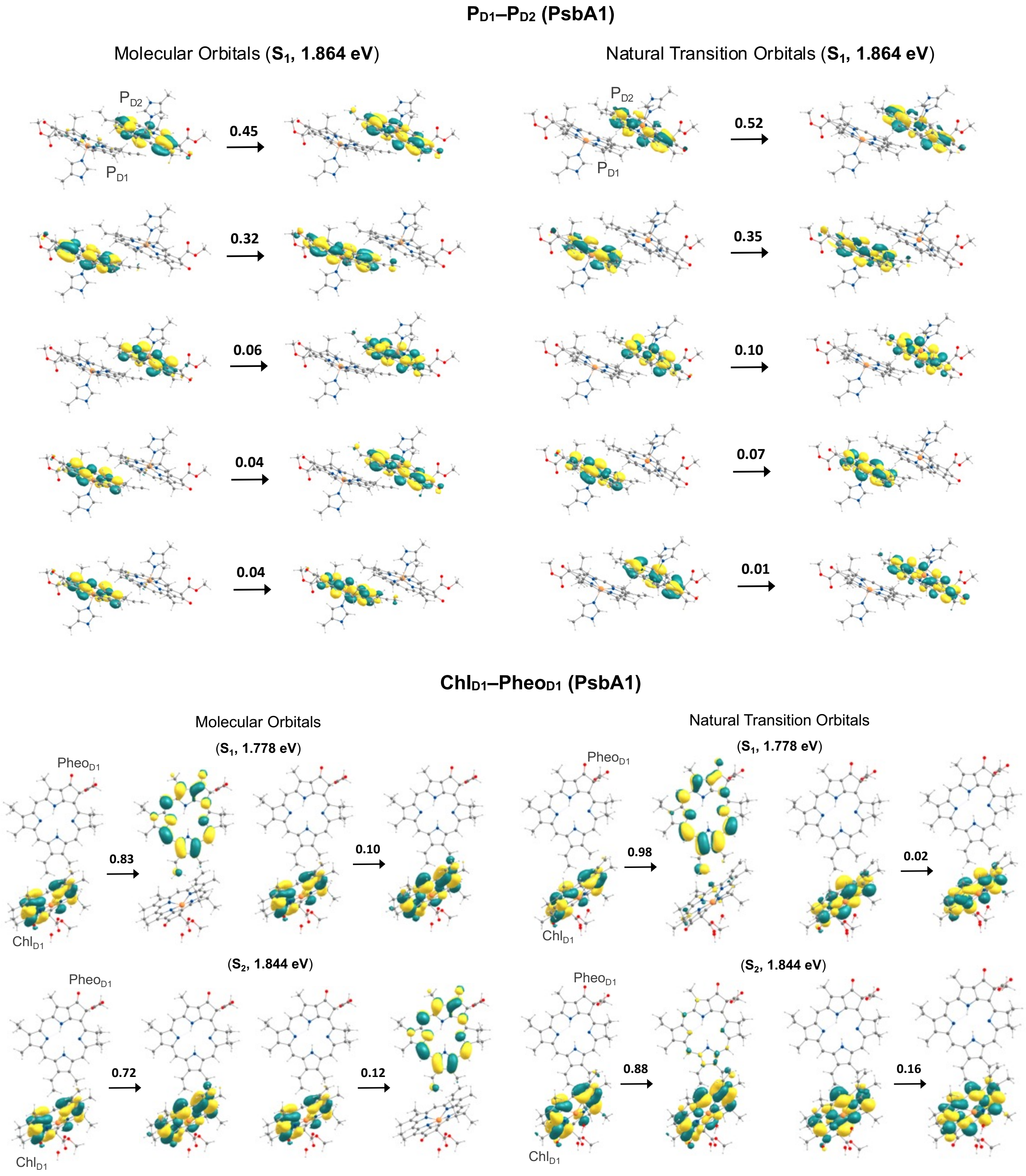

Fig. S14. Identity and nature of one selected excited state (S1, snapshot 1) for the P_{D1}–P_{D2} pair (top) and two selected excited states (S1 and S2, snapshot 21) for the Chl_{D1}–$Pheo_{D1}$ pair (bottom) in terms of canonical molecular orbitals (MOs, left) and natural transition orbitals (NTOs, right) and relative contributions to a given excitation obtained from ωB97X-D3(BJ) TD-DFT calculations, on the PsbA1 variant. Note that the molecular orbitals are universal for each system and all excitations, whereas the NTOs are unique to each individual excitation.

PsbA1

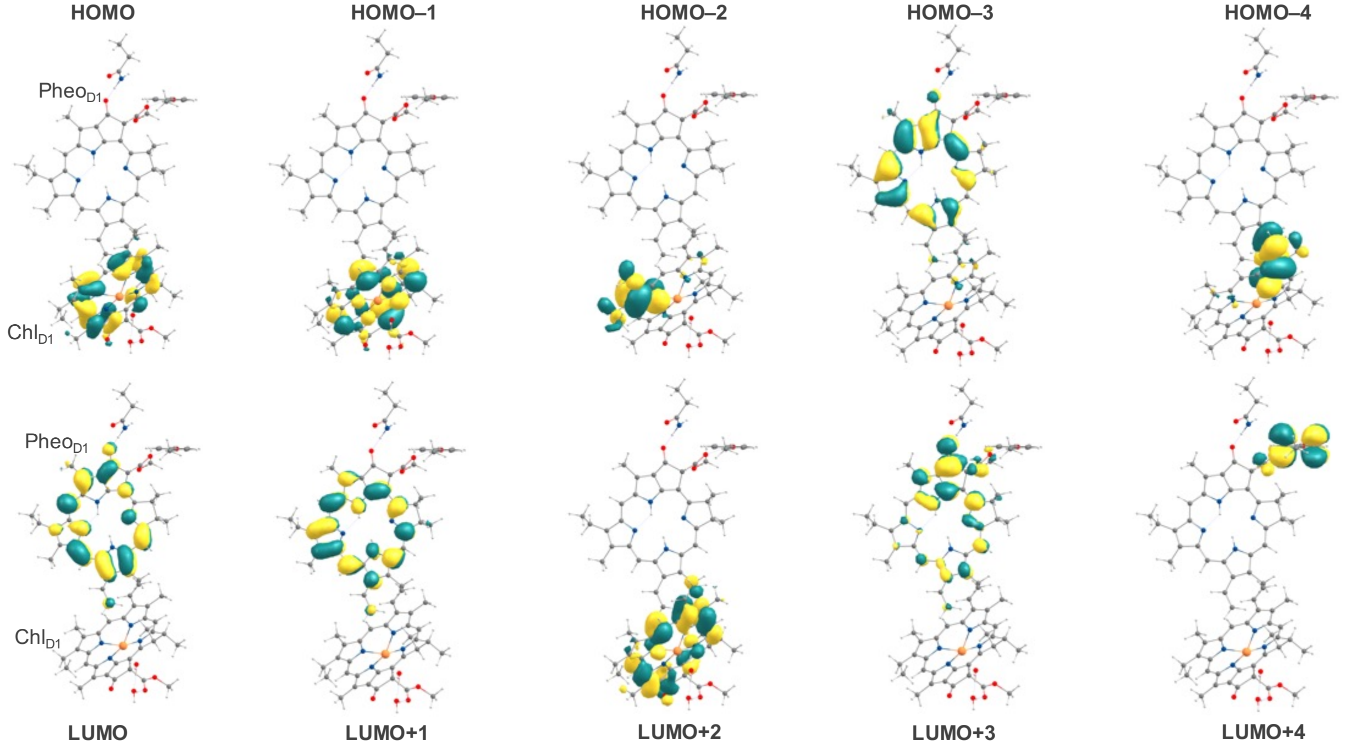

PsbA2-GLU(H)

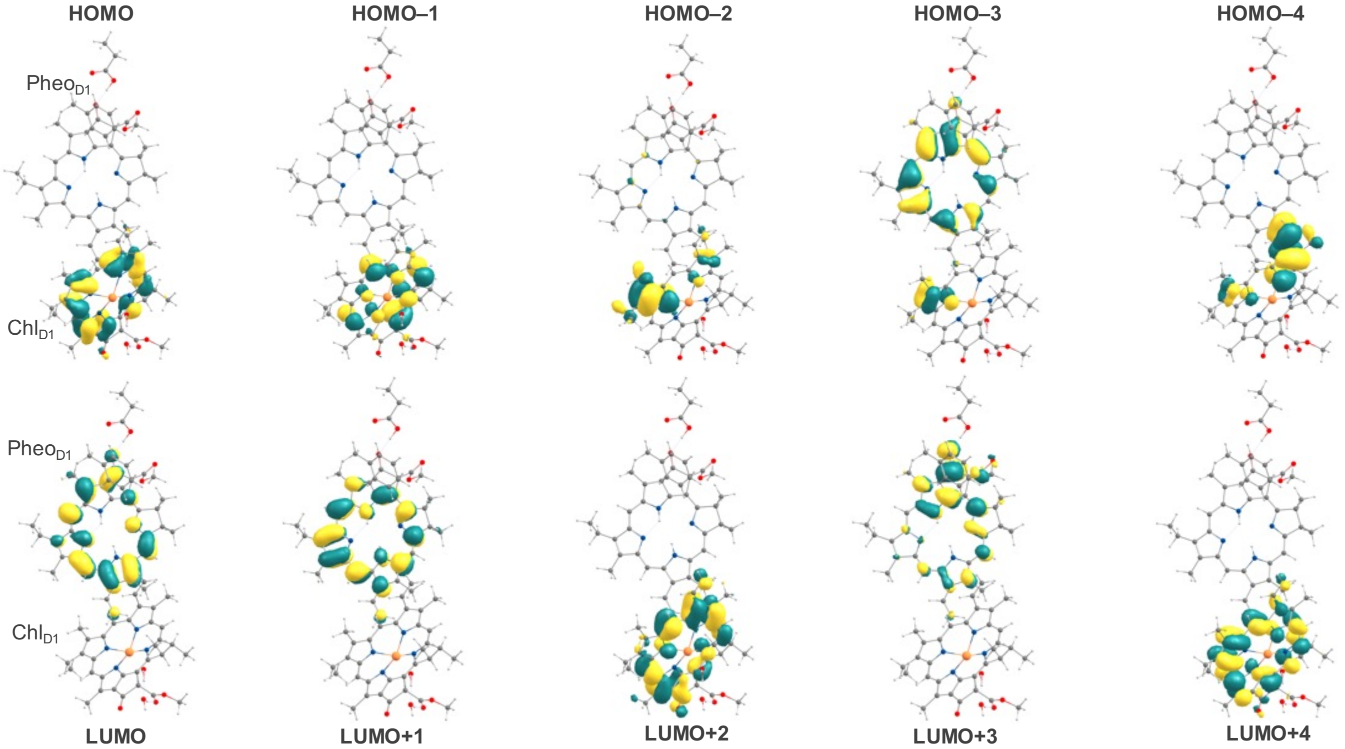

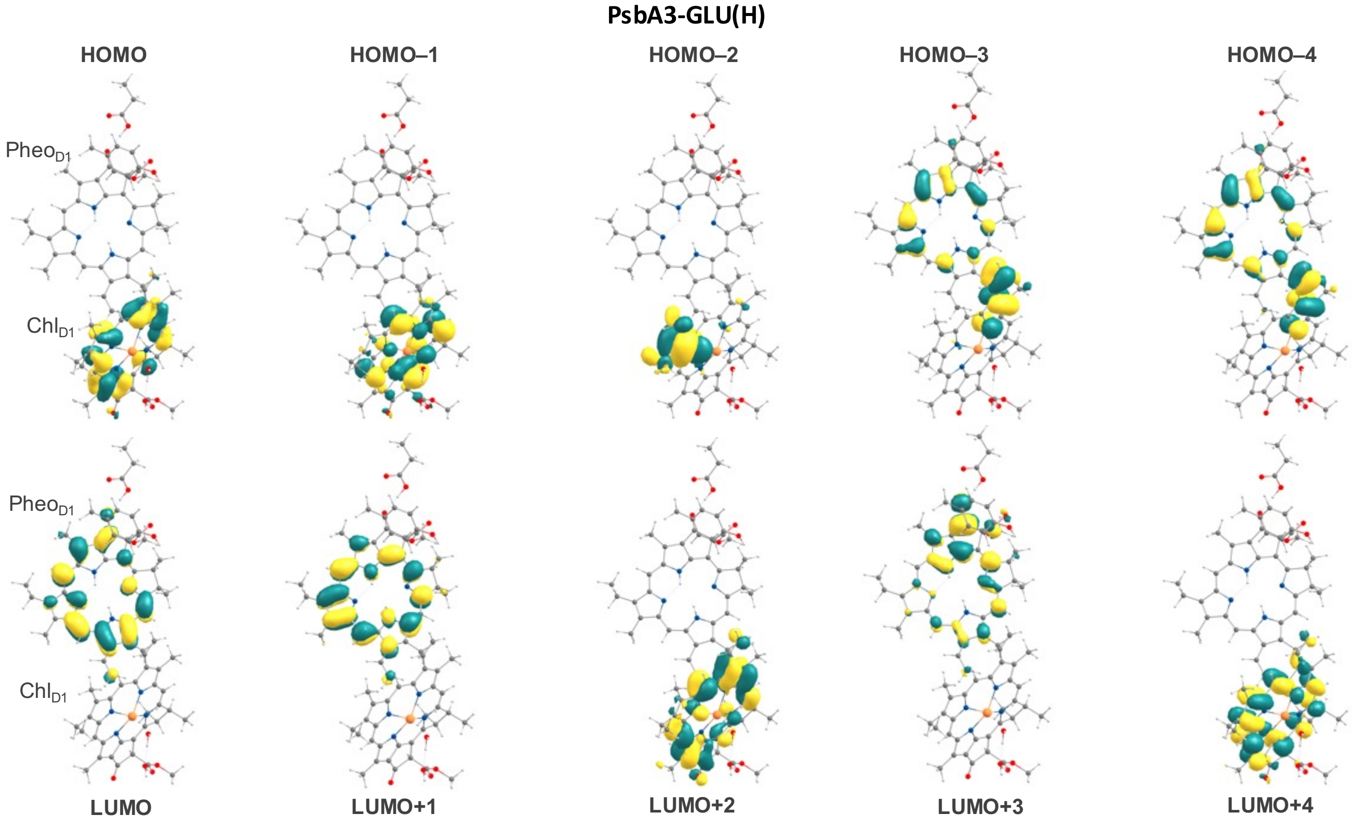

Fig. S15. The frontier molecular orbitals (HOMO–4 to LUMO+4) of the **Chl_{D1}–$Pheo_{D1}$** pair in each PsbA variant, calculated using TD-DFT and QM/MM, shown here for snapshot 1 in each case.

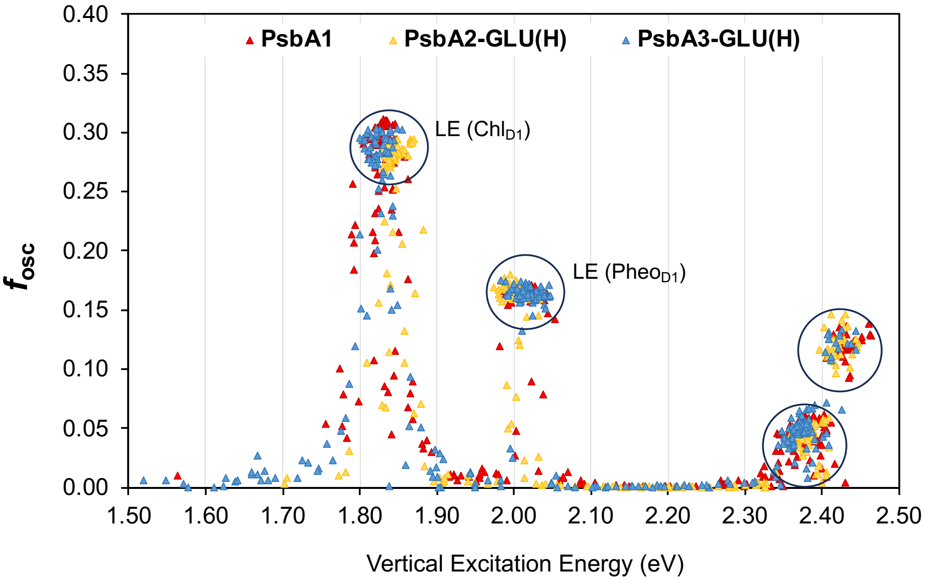

Fig. S16. Distribution of vertical excitation energies (S1 to S5) vs oscillator strengths (**f_{osc}**) of the Chl_{D1}–$Pheo_{D1}$ pair in each PsbA variant, calculated using TD-DFT and QM/MM. The corresponding values are listed in Table S8. The encircled clusters indicate f_{osc} corresponding to major local excitation (LE) character, while the f_{osc} ~ 0.0 indicate a high CT contribution. The spread in f_{osc} at lower energies can be attributed to a mixed LE-charge transfer character. It is to be noted that the extent of this mixing is dynamic and depends largely on the geometric structure of both pigments and changes within the surrounding protein matrix.

Supplementary Tables

Table S1. Location of all variant residues that are located more than 15 Å away from any cofactor in RC within the D1 copies of PsbA1, PsbA2 and PsbA3 variants.

Residue position	PsbA1	PsbA2	PsbA3
11	Ala	Ala	Leu
16	Arg	Arg	Gln
19	Asn	Asp	Ser
21	Val	Ile	Val
25	Asp	Glu	Asp
30	Val	Ile	Val
36	Ile	Ile	Leu
93	Phe	Phe	Leu
98	Glu	Asp	Glu
155	Phe	Thr	Thr
233	Ala	Thr	Ala
307	Ile	Val	Val
310	Lys	Gln	Glu
348	Ser	Leu	Ser
359	Asp	Glu	Asp

Table S2. Average center-to-center distances (in Å) of RC pigments (Chl_{D1}–$Pheo_{D1}$, Chl_{D2}–$Pheo_{D2}$, P_{D1}–P_{D2}) along 60 ns of production MD simulations for PsbA1-A2-A3 variants.

Pigments	PsbA1	PsbA2-Glu(H)	PsbA2-Glu	PsbA3-Glu(H)	PsbA3-Glu
Chl_{D1}–$Pheo_{D1}$	9.62±0.21	9.76±0.20	9.67±0.21	9.77±0.21	9.69±0.21
P_{D1}–P_{D2}	8.43±0.21	8.42±0.22	8.38±0.21	8.32±0.21	8.39±0.22
Chl_{D2}–$Pheo_{D2}$	9.70±0.23	9.75±0.26	9.76±0.23	9.82±0.25	9.81±0.27

Table S3. Energy Decomposition of the $Pheo_{D1}$ binding energy (in kcal mol^{-1}) in terms of VdW, electrostatic, non-polar, dispersion and solvation terms computed using Generalized Born (MM-GBSA) and Poisson Boltzmann (MM-PBSA) approaches respectively. The binding energies were computed using the first 60 ns of production MD over an ensemble of 300 equidistant snapshots.

**GB Energy term**	**PsbA1**	**PsbA2-Glu(H)**	**PsbA2-Glu**	**PsbA3-Glu(H)**	**PsbA3-Glu**
vdW	-94.99	-95.27	-95.83	-95.71	-94.71
E_{EL}	-12.40	-9.75	-2.29	-12.20	-4.17
E_{PB}	25.04	23.30	23.73	25.71	23.87
E_{NPOLAR}	-67.46	-66.98	-66.99	-67.61	-66.62
E_{DISPER}	114.93	114.37	114.56	114.95	114.56
ΔG gas	-107.39	-105.02	-98.12	-107.91	-98.88
ΔG solv	72.52	70.70	71.29	73.06	71.81
**ΔG total**	-34.88	-34.33	-26.83	-34.85	-27.07

**PB Energy term**	**PsbA1**	**PsbA2-Glu(H)**	**PsbA2-Glu**	**PsbA3-Glu(H)**	**PsbA3-Glu**
vdW	-94.99	-95.27	-95.83	-95.71	-94.71
E_{EL}	-24.80	-19.50	-4.59	-24.39	-8.34
E_{GB}	49.59	45.27	33.45	49.85	37.50
E_{SURF}	-11.56	-11.41	-11.40	-11.56	-11.42
ΔG gas	-119.79	-114.77	-100.42	-120.11	-103.05
ΔG solv	38.03	33.86	22.05	38.29	26.08
**ΔG total**	-81.76	-80.90	-78.37	-81.81	-76.97

Table S4. Excited state properties of the $Chl_{D1}Pheo_{D1}$ pair in each PsbA variant, computed using TDDFT with QM/MM at the ωB97X-D3BJ/def2-TZVP level of theory. The nature of the transitions are indicated as local excitation (LE) or charge-transfer (CT), based on natural transition orbitals (NTOs); f_{osc} are the corresponding oscillator strengths for each state.

PsbA1 (snapshot 1)			
State	NTO contribution	f_{osc}	Energy (eV)
1	LE (Chl_{D1}) + CT ($Chl_{D1}^{\delta+}Pheo_{D1}^{\delta-}$), 0.89<br>LE ($Chl_{D1}$), 0.14	0.27	1.806
2	CT ($Chl_{D1}^{\delta+}Pheo_{D1}^{\delta-}$) + LE ($Chl_{D1}$), 0.97	0.04	1.885
3	LE ($Pheo_{D1}$), 0.80<br>LE ($Pheo_{D1}$), 0.24	0.17	2.048
4	LE (Chl_{D1}) + CT ($Chl_{D1}^{\delta+}Pheo_{D1}^{\delta-}$), 0.87<br>LE ($Chl_{D1}$), 0.14	0.04	2.346
5	LE (Chl_{D1}) + CT ($Chl_{D1}^{\delta+}Pheo_{D1}^{\delta-}$), 0.88<br>LE ($Chl_{D1}$), 0.12	0.02	2.392
6	LE ($Pheo_{D1}$), 0.79<br>LE ($Pheo_{D1}$), 0.23	0.19	2.447
7	CT ($Chl_{D1}^{\delta+}Pheo_{D1}^{\delta-}$), 0.998	0.00	3.038

PsbA2-Glu(H) (snapshot 1)			
State	NTO contribution	f_{osc}	Energy (eV)
1	LE (Chl_{D1}), 0.87<br>LE (Chl_{D1}), 0.16	0.28	1.816
2	CT ($Chl_{D1}^{\delta+}Pheo_{D1}^{\delta-}$), 0.97	0.02	1.932
3	LE ($Pheo_{D1}$), 0.80<br>LE ($Pheo_{D1}$), 0.23	0.17	2.029
4	LE (Chl_{D1}) + CT ($Chl_{D1}^{\delta+}Pheo_{D1}^{\delta-}$), 0.80<br>LE ($Chl_{D1}$), 0.22	0.04	2.343
5	CT ($Chl_{D1}^{\delta+}Pheo_{D1}^{\delta-}$), 0.91<br>LE ($Chl_{D1}$), 0.06	0.02	2.415
6	LE ($Pheo_{D1}$), 0.79<br>LE ($Pheo_{D1}$), 0.21	0.16	2.431
7	CT ($Chl_{D1}^{\delta+}Pheo_{D1}^{\delta-}$), 0.997	0.00	3.061

PsbA2-Glu (snapshot 1)			
State	NTO contribution	f_{osc}	Energy (eV)
1	LE (Chl_{D1}), 0.55<br>LE ($Pheo_{D1}$), 0.30<br>LE (Chl_{D1}), 0.11	0.40	1.877
2	LE ($Pheo_{D1}$), 0.50<br>LE (Chl_{D1}), 0.31<br>LE ($Pheo_{D1}$), 0.17	0.06	1.904
3	LE (Chl_{D1}), 0.66<br>LE (Chl_{D1}), 0.37	0.04	2.360
4	LE ($Pheo_{D1}$), 0.77<br>LE ($Pheo_{D1}$), 0.25	0.07	2.412
5	CT ($Chl_{D1}^{\delta+}Pheo_{D1}^{\delta-}$), 0.997	0.01	2.735
6	CT ($Chl_{D1}^{\delta+}Pheo_{D1}^{\delta-}$), 0.995	0.00	3.148

PsbA3-Glu(H) (snapshot 1)			
State	NTO contribution	f_{osc}	Energy (eV)
1	CT ($Chl_{D1}^{\delta+}Pheo_{D1}^{\delta-}$), 0.99	0.04	1.701
2	LE (Chl_{D1}), 0.88<br>LE (Chl_{D1}), 0.16	0.27	1.819
3	LE ($Pheo_{D1}$), 0.80<br>LE ($Pheo_{D1}$), 0.23	0.18	2.027

4	CT ($Chl_{D1}^{\delta+}Pheo_{D1}^{\delta-}$), 0.99	0.00	2.223
5	LE (Chl_{D1}), 0.76<br>LE (Chl_{D1}), 0.27	0.05	2.368
6	LE ($Pheo_{D1}$), 0.80<br>LE ($Pheo_{D1}$), 0.22	0.19	2.427
7	CT ($Chl_{D1}^{\delta+}Pheo_{D1}^{\delta-}$), 0.998	0.00	2.896

**PsbA3-Glu (snapshot 1)**			
**State**	**NTO contribution**	f_{osc}	**Energy (eV)**
1	LE (Chl_{D1}), 0.67<br>LE ($Pheo_{D1}$), 0.19<br>LE (Chl_{D1}), 0.13	0.42	1.853
2	LE ($Pheo_{D1}$), 0.61<br>LE (Chl_{D1}), 0.21<br>LE ($Pheo_{D1}$), 0.20	0.07	1.889
3	LE (Chl_{D1}), 0.67<br>LE (Chl_{D1}), 0.36	0.05	2.343
4	LE ($Pheo_{D1}$), 0.78<br>LE ($Pheo_{D1}$), 0.24	0.07	2.388
5	CT ($Chl_{D1}^{\delta+}Pheo_{D1}^{\delta-}$), 0.998	0.00	2.701
6	CT ($Chl_{D1}^{\delta+}Pheo_{D1}^{\delta-}$), 0.994	0.00	3.140

Table S5. Excited state properties of Chl_{D1}–$Pheo_{D1}$ in each PsbA variant, computed on the QM/MM optimized geometries of the pair embedded within the protein matrix for “crystal-like” snapshot 1. All energy values are reported in eV.

**Major NTO character**	**PsbA1-Gln**	**PsbA2-Glu**		**PsbA3-Glu**		**PsbA2-Glu(H)**		**PsbA3-Glu(H)**	
LE (Chl_{D1})	1.806	1.877	0.071	1.853	0.047	1.816	0.01	1.819	0.013
CT ($Chl_{D1}^{\delta+}Pheo_{D1}^{\delta-}$)	1.885	2.735	0.85	2.701	0.816	1.932	0.047	1.701	-0.184
LE ($Pheo_{D1}$)	2.048	1.904	-0.144	1.889	-0.159	2.029	-0.019	2.027	-0.021
LE (Chl_{D1})	2.346	2.36	0.014	2.343	-0.003	2.343	-0.003	2.368	0.022
CT ($Chl_{D1}^{\delta+}Pheo_{D1}^{\delta-}$)	2.392	3.148	0.763	3.140	0.748	2.415	0.023	2.223	-0.169
LE ($Pheo_{D1}$)	2.447	2.412	-0.035	2.388	-0.059	2.431	-0.016	2.427	0.020

Table S6. Excited state properties of P_{D1}–P_{D2} in each PsbA variant, computed on the QM/MM optimized geometries of the pair embedded within the protein matrix for “crystal-like” snapshot 1. All energy values are reported in eV.

**State**	**NTO character**	**PsbA1-Gln**	**PsbA2-Glu(H)**	**PsbA3-Glu(H)**
1	LE (P_{D2}) + LE (P_{D1})	1.864	1.859	1.868
2	LE (P_{D1}) + LE (P_{D2})	1.884	1.891	1.897
3	LE (P_{D2})	2.403	2.404	2.426
4	LE (P_{D1})	2.422	2.434	2.442
5	CT ($P_{D1}^{\delta+}P_{D2}^{\delta-}$)	2.911	3.031	2.870

Table S7. QM(TD-DFT)/MM vertical excitation energies and corresponding oscillator strengths of Chl_{D1}–Pheo_{D1} in each PsbA variant, computed on the QM/MM optimized geometries of Chl_{D1}–Pheo_{D1} embedded within the protein matrix for 60 independent snapshots.

	**PsbA1**			**PsbA2-Glu(H)**			**PsbA3-Glu(H)**		
**State**	**Wavelength (nm)**	**Energy (eV)**	f_{osc}	**Wavelength (nm)**	**Energy (eV)**	f_{osc}	**Wavelength (nm)**	**Energy (eV)**	f_{osc}
1	698.7	1.774	0.10	672.3	1.844	0.28	743.5	1.668	0.03
2	681.5	1.819	0.20	647.3	1.915	0.01	680.5	1.822	0.28
3	617.0	2.009	0.17	617.5	2.008	0.17	609.3	2.035	0.17
4	535.7	2.314	0.00	523.0	2.371	0.04	567.0	2.187	0.00
5	525.2	2.361	0.04	517.0	2.398	0.02	519.9	2.385	0.05
1	692.4	1.791	0.21	693.7	1.787	0.03	682.9	1.816	0.30
2	676.6	1.832	0.09	670.8	1.848	0.25	637.5	1.945	0.01
3	617.6	2.008	0.17	618.3	2.005	0.17	613.9	2.020	0.17
4	528.8	2.345	0.02	543.2	2.282	0.00	522.5	2.373	0.05
5	523.4	2.369	0.03	521.0	2.380	0.04	514.5	2.410	0.13
1	690.9	1.795	0.22	671.9	1.845	0.29	683.8	1.813	0.28
2	674.8	1.837	0.08	622.8	1.991	0.09	649.2	1.910	0.02
3	619.8	2.000	0.16	618.9	2.003	0.08	613.6	2.021	0.17
4	528.5	2.346	0.01	521.9	2.376	0.05	524.7	2.363	0.04
5	522.8	2.372	0.04	515.1	2.407	0.12	515.5	2.405	0.11
1	683.8	1.813	0.29	726.8	1.706	0.01	680.2	1.823	0.30
2	643.2	1.928	0.01	672.9	1.843	0.27	637.5	1.945	0.01
3	621.3	1.996	0.16	614.3	2.018	0.16	613.7	2.020	0.17
4	525.1	2.361	0.05	569.7	2.176	0.00	520.1	2.384	0.06
5	510.7	2.428	0.12	524.8	2.363	0.05	514.7	2.409	0.13
1	692.1	1.791	0.26	679.4	1.825	0.16	688.4	1.801	0.21
2	673.1	1.842	0.04	667.0	1.859	0.13	664.5	1.866	0.09
3	616.5	2.011	0.17	619.0	2.003	0.17	614.4	2.018	0.17
4	529.8	2.340	0.03	533.0	2.326	0.00	530.4	2.338	0.01
5	524.0	2.366	0.02	519.8	2.385	0.04	516.1	2.402	0.04
1	681.4	1.820	0.11	671.4	1.847	0.29	684.8	1.811	0.30
2	665.3	1.864	0.18	627.7	1.975	0.17	637.7	1.944	0.01
3	610.6	2.031	0.16	549.7	2.255	0.00	614.9	2.016	0.17
4	540.9	2.292	0.01	521.1	2.379	0.05	523.6	2.368	0.05
5	515.2	2.407	0.05	514.0	2.412	0.11	515.0	2.407	0.13
1	679.1	1.826	0.25	671.2	1.847	0.29	682.5	1.817	0.27
2	658.8	1.882	0.04	622.0	1.993	0.05	651.0	1.905	0.03
3	612.7	2.024	0.17	617.9	2.007	0.12	614.6	2.017	0.17
4	529.7	2.341	0.02	520.8	2.381	0.05	524.9	2.362	0.03
5	518.1	2.393	0.04	514.3	2.411	0.13	514.6	2.409	0.04
1	688.9	1.800	0.07	670.2	1.850	0.29	684.6	1.811	0.28
2	670.0	1.851	0.22	624.4	1.986	0.17	653.1	1.898	0.03
3	606.9	2.043	0.16	585.6	2.117	0.00	615.9	2.013	0.17
4	546.2	2.270	0.01	518.8	2.390	0.06	525.6	2.359	0.04
5	518.6	2.391	0.05	515.0	2.407	0.11	515.2	2.407	0.05
1	680.8	1.821	0.21	677.7	1.829	0.07	721.0	1.720	0.01
2	664.4	1.866	0.08	668.1	1.856	0.21	675.0	1.837	0.28
3	612.7	2.024	0.17	613.6	2.021	0.17	611.1	2.029	0.17
4	533.5	2.324	0.01	535.5	2.315	0.00	560.6	2.212	0.00
5	516.4	2.401	0.04	520.3	2.383	0.04	520.7	2.381	0.05
1	675.8	1.835	0.28	675.6	1.835	0.18	677.3	1.831	0.29
2	644.7	1.923	0.01	667.3	1.858	0.11	651.8	1.902	0.01
3	611.5	2.028	0.17	616.5	2.011	0.17	619.6	2.001	0.17
4	521.1	2.379	0.04	532.3	2.329	0.00	524.8	2.363	0.03
5	513.0	2.417	0.02	519.9	2.385	0.04	519.1	2.388	0.02
1	665.3	1.864	0.26	675.4	1.836	0.28	678.9	1.826	0.28
2	632.9	1.959	0.02	649.9	1.908	0.00	654.3	1.895	0.01
3	608.7	2.037	0.16	615.5	2.014	0.17	612.8	2.023	0.17
4	517.0	2.398	0.04	523.1	2.370	0.04	527.1	2.352	0.04
5	508.6	2.438	0.09	518.2	2.393	0.01	520.4	2.382	0.01
1	671.8	1.846	0.27	671.6	1.846	0.28	676.4	1.833	0.30
2	626.7	1.978	0.01	620.3	1.999	0.17	625.1	1.983	0.01
3	611.2	2.029	0.16	591.2	2.097	0.00	615.4	2.015	0.17
4	516.9	2.399	0.05	522.6	2.372	0.04	522.0	2.375	0.05

5	508.9	2.436	0.12	511.6	2.423	0.12	507.1	2.445	0.13
1	667.2	1.858	0.28	698.9	1.774	0.01	672.0	1.845	0.29
2	620.5	1.998	0.16	671.6	1.846	0.28	618.1	2.006	0.16
3	600.7	2.064	0.01	615.5	2.014	0.17	604.9	2.050	0.00
4	515.5	2.405	0.05	555.4	2.232	0.00	521.2	2.379	0.06
5	509.9	2.432	0.11	523.1	2.370	0.05	512.6	2.419	0.12
1	666.6	1.860	0.28	673.7	1.840	0.29	673.5	1.841	0.30
2	619.3	2.002	0.17	619.8	2.000	0.17	622.1	1.993	0.16
3	578.1	2.145	0.00	602.5	2.058	0.00	568.7	2.180	0.00
4	513.9	2.413	0.05	522.9	2.371	0.05	522.7	2.372	0.06
5	508.9	2.436	0.12	512.1	2.421	0.12	513.9	2.413	0.11
1	669.9	1.851	0.28	672.2	1.844	0.28	675.2	1.836	0.30
2	617.9	2.007	0.16	648.8	1.911	0.01	618.3	2.005	0.16
3	600.3	2.065	0.01	615.2	2.015	0.17	588.1	2.108	0.00
4	515.8	2.404	0.06	521.8	2.376	0.03	521.8	2.376	0.05
5	508.3	2.439	0.12	517.5	2.396	0.02	509.5	2.433	0.12
1	677.5	1.830	0.29	672.1	1.845	0.29	676.5	1.833	0.28
2	635.3	1.952	0.01	625.1	1.983	0.18	655.3	1.892	0.01
3	608.3	2.038	0.17	577.3	2.148	0.00	617.6	2.008	0.17
4	525.5	2.359	0.05	521.8	2.376	0.05	524.0	2.366	0.02
5	515.1	2.407	0.01	513.5	2.414	0.12	518.1	2.393	0.03
1	674.0	1.840	0.29	695.7	1.782	0.01	675.3	1.836	0.28
2	622.8	1.991	0.01	673.9	1.840	0.27	616.8	2.010	0.17
3	610.4	2.031	0.16	618.5	2.005	0.17	549.9	2.255	0.00
4	521.7	2.377	0.06	547.2	2.266	0.00	522.2	2.374	0.05
5	505.5	2.453	0.12	520.6	2.382	0.04	507.1	2.445	0.12
1	671.0	1.848	0.29	728.1	1.703	0.00	702.0	1.766	0.02
2	612.5	2.024	0.17	674.9	1.837	0.29	677.6	1.830	0.26
3	564.7	2.196	0.00	617.7	2.007	0.17	608.4	2.038	0.17
4	519.3	2.388	0.06	563.5	2.200	0.00	550.7	2.251	0.00
5	503.0	2.465	0.13	519.4	2.387	0.04	523.1	2.370	0.05
1	672.1	1.845	0.29	677.1	1.831	0.29	756.5	1.639	0.00
2	612.6	2.024	0.09	653.6	1.897	0.01	681.8	1.818	0.28
3	608.0	2.039	0.08	621.0	1.997	0.18	612.5	2.024	0.17
4	517.6	2.395	0.06	521.7	2.377	0.03	577.8	2.146	0.00
5	503.7	2.461	0.14	517.5	2.396	0.01	522.7	2.372	0.04
1	674.3	1.839	0.30	674.9	1.837	0.29	693.6	1.788	0.09
2	613.0	2.023	0.17	626.2	1.980	0.01	680.1	1.823	0.20
3	576.7	2.150	0.00	618.7	2.004	0.17	614.2	2.019	0.17
4	522.8	2.372	0.06	520.9	2.380	0.05	539.3	2.299	0.00
5	505.7	2.452	0.13	510.0	2.431	0.15	523.2	2.370	0.05
1	697.5	1.778	0.05	671.1	1.847	0.29	741.5	1.672	0.01
2	672.4	1.844	0.25	625.8	1.981	0.17	679.9	1.824	0.28
3	617.0	2.009	0.17	564.5	2.196	0.00	608.8	2.037	0.17
4	545.2	2.274	0.00	522.1	2.375	0.05	573.4	2.162	0.00
5	519.2	2.388	0.05	514.9	2.408	0.12	522.6	2.372	0.05
1	679.1	1.826	0.29	671.3	1.847	0.29	788.7	1.572	0.00
2	644.8	1.923	0.01	625.8	1.981	0.16	680.4	1.822	0.28
3	621.7	1.994	0.17	608.6	2.037	0.00	609.2	2.035	0.17
4	525.9	2.358	0.04	520.1	2.384	0.04	598.8	2.071	0.00
5	514.7	2.409	0.11	517.1	2.398	0.12	523.0	2.371	0.05
1	680.5	1.822	0.30	672.0	1.845	0.29	679.0	1.826	0.28
2	633.6	1.957	0.01	623.8	1.988	0.17	635.6	1.951	0.00
3	620.3	1.999	0.16	611.1	2.029	0.00	613.2	2.022	0.17
4	525.9	2.358	0.05	518.9	2.389	0.05	523.5	2.368	0.05
5	513.4	2.415	0.13	515.7	2.404	0.14	510.9	2.427	0.07
1	677.5	1.830	0.27	671.3	1.847	0.29	737.3	1.682	0.01
2	654.8	1.893	0.03	622.8	1.991	0.17	679.9	1.824	0.27
3	617.7	2.007	0.17	579.3	2.140	0.00	606.0	2.046	0.17
4	524.6	2.363	0.03	518.4	2.392	0.05	570.8	2.172	0.00
5	516.6	2.400	0.02	511.5	2.424	0.13	523.6	2.368	0.05
1	681.2	1.820	0.27	664.4	1.866	0.29	762.3	1.626	0.01
2	658.5	1.883	0.03	625.5	1.982	0.16	680.2	1.823	0.30
3	616.5	2.011	0.17	563.2	2.201	0.00	607.5	2.041	0.15
4	528.1	2.348	0.03	516.6	2.400	0.06	588.6	2.106	0.00
5	519.9	2.385	0.02	514.8	2.408	0.11	525.2	2.361	0.06
1	691.5	1.793	0.21	667.7	1.857	0.28	743.3	1.668	0.01
2	671.8	1.846	0.09	638.7	1.941	0.01	680.7	1.821	0.29
3	607.7	2.040	0.16	620.3	1.999	0.16	612.1	2.026	0.16
4	533.2	2.325	0.02	521.4	2.378	0.03	575.8	2.153	0.00
5	524.2	2.365	0.04	514.5	2.410	0.03	525.8	2.358	0.05

1	684.6	1.811	0.30	664.1	1.867	0.29	679.0	1.826	0.30
2	617.4	2.008	0.17	622.3	1.992	0.16	630.3	1.967	0.01
3	571.8	2.168	0.00	567.5	2.185	0.00	613.9	2.020	0.16
4	529.4	2.342	0.05	516.2	2.402	0.06	522.8	2.372	0.06
5	508.4	2.439	0.12	513.4	2.415	0.13	510.9	2.427	0.13
1	686.8	1.805	0.29	662.5	1.871	0.29	688.2	1.802	0.15
2	646.5	1.918	0.01	622.3	1.992	0.16	673.2	1.842	0.15
3	611.6	2.027	0.16	552.5	2.244	0.00	611.2	2.029	0.16
4	528.5	2.346	0.05	514.5	2.410	0.06	536.0	2.313	0.00
5	509.9	2.432	0.00	510.2	2.430	0.13	521.6	2.377	0.05
1	691.3	1.793	0.18	665.4	1.863	0.28	685.0	1.810	0.15
2	671.7	1.846	0.12	624.6	1.985	0.16	670.2	1.850	0.15
3	611.9	2.026	0.16	581.5	2.132	0.00	610.7	2.030	0.16
4	534.1	2.321	0.01	519.9	2.385	0.04	536.6	2.311	0.00
5	524.1	2.366	0.03	512.7	2.418	0.11	520.5	2.382	0.05
1	792.9	1.564	0.01	675.7	1.835	0.07	746.7	1.660	0.01
2	682.3	1.817	0.29	658.5	1.883	0.22	681.3	1.820	0.29
3	606.4	2.045	0.15	613.0	2.023	0.16	609.7	2.034	0.16
4	594.1	2.087	0.01	538.8	2.301	0.00	575.5	2.154	0.00
5	524.1	2.366	0.05	514.0	2.412	0.15	524.5	2.364	0.05
1	679.5	1.825	0.24	664.4	1.866	0.29	705.1	1.758	0.04
2	663.3	1.869	0.06	619.9	2.000	0.16	677.1	1.831	0.27
3	613.6	2.021	0.16	571.5	2.169	0.00	613.9	2.020	0.16
4	532.6	2.328	0.01	515.9	2.403	0.05	552.4	2.244	0.00
5	521.3	2.378	0.04	511.7	2.423	0.12	524.7	2.363	0.05
1	678.2	1.828	0.29	663.3	1.869	0.29	800.3	1.549	0.01
2	646.9	1.917	0.01	618.9	2.003	0.17	684.4	1.812	0.30
3	615.2	2.015	0.16	556.1	2.230	0.00	607.2	2.042	0.15
4	527.0	2.353	0.04	515.4	2.406	0.06	603.0	2.056	0.01
5	518.8	2.390	0.01	509.3	2.434	0.14	523.7	2.367	0.05
1	676.9	1.832	0.30	677.0	1.831	0.27	872.6	1.421	0.00
2	625.9	1.981	0.01	651.1	1.904	0.01	687.1	1.804	0.29
3	616.4	2.011	0.16	613.9	2.020	0.17	635.7	1.950	0.00
4	524.2	2.365	0.05	524.8	2.363	0.04	617.2	2.009	0.16
5	507.5	2.443	0.12	516.5	2.400	0.00	525.6	2.359	0.04
1	695.1	1.784	0.04	675.3	1.836	0.28	746.9	1.660	0.01
2	672.3	1.844	0.25	619.5	2.001	0.17	686.1	1.807	0.29
3	615.1	2.016	0.16	601.2	2.062	0.00	620.5	1.998	0.17
4	545.8	2.272	0.00	525.3	2.360	0.04	565.6	2.192	0.00
5	520.3	2.383	0.04	508.9	2.436	0.12	524.5	2.364	0.04
1	680.9	1.821	0.23	685.1	1.810	0.11	815.8	1.520	0.01
2	665.3	1.864	0.07	673.8	1.840	0.17	684.7	1.811	0.28
3	614.3	2.018	0.17	615.3	2.015	0.17	617.3	2.008	0.16
4	528.6	2.346	0.01	535.8	2.314	0.00	602.6	2.057	0.00
5	516.5	2.400	0.04	525.4	2.360	0.03	525.9	2.358	0.03
1	679.7	1.824	0.26	672.4	1.844	0.28	942.9	1.315	0.00
2	656.7	1.888	0.04	621.2	1.996	0.17	686.6	1.806	0.30
3	617.8	2.007	0.16	596.0	2.080	0.00	674.5	1.838	0.00
4	525.7	2.358	0.02	523.9	2.367	0.04	616.9	2.010	0.16
5	516.9	2.399	0.03	513.0	2.417	0.11	525.0	2.362	0.04
1	681.2	1.820	0.30	673.3	1.841	0.28	890.5	1.392	0.00
2	641.9	1.932	0.01	623.9	1.987	0.17	686.4	1.806	0.29
3	620.0	2.000	0.17	572.4	2.166	0.00	650.6	1.906	0.00
4	520.9	2.380	0.05	525.5	2.359	0.04	617.3	2.008	0.16
5	510.6	2.428	0.12	512.6	2.419	0.10	527.3	2.351	0.04
1	696.9	1.779	0.08	673.8	1.840	0.27	852.3	1.455	0.00
2	673.4	1.841	0.23	621.1	1.996	0.17	683.6	1.814	0.29
3	618.1	2.006	0.17	558.8	2.219	0.00	625.0	1.984	0.00
4	538.5	2.302	0.00	526.8	2.354	0.04	617.0	2.009	0.16
5	515.2	2.407	0.05	513.4	2.415	0.11	524.6	2.363	0.04
1	676.7	1.832	0.31	671.6	1.846	0.28	905.8	1.369	0.00
2	622.1	1.993	0.15	619.6	2.001	0.17	688.7	1.800	0.30
3	610.1	2.032	0.01	565.7	2.192	0.00	651.7	1.902	0.00
4	515.7	2.404	0.05	524.4	2.364	0.05	616.0	2.013	0.16
5	510.2	2.430	0.12	511.3	2.425	0.11	526.3	2.356	0.04
1	705.9	1.756	0.05	674.2	1.839	0.27	739.2	1.677	0.01
2	676.7	1.832	0.25	635.6	1.951	0.00	683.7	1.813	0.28
3	616.0	2.013	0.17	619.8	2.000	0.17	618.7	2.004	0.16
4	546.3	2.270	0.00	526.9	2.353	0.04	564.7	2.196	0.00
5	520.3	2.383	0.04	512.7	2.418	0.10	527.6	2.350	0.04

1	672.7	1.843	0.31	674.0	1.840	0.28	680.9	1.821	0.27
2	619.1	2.003	0.17	649.1	1.910	0.01	652.4	1.900	0.02
3	581.2	2.133	0.00	613.7	2.020	0.17	613.7	2.020	0.16
4	522.1	2.375	0.06	520.8	2.381	0.03	529.0	2.344	0.04
5	511.5	2.424	0.14	515.3	2.406	0.01	520.9	2.380	0.02
1	675.5	1.835	0.31	674.3	1.839	0.28	691.1	1.794	0.12
2	623.0	1.990	0.17	614.6	2.017	0.14	673.6	1.841	0.17
3	594.2	2.087	0.00	610.0	2.033	0.03	607.4	2.041	0.16
4	521.3	2.378	0.06	523.3	2.369	0.04	544.1	2.279	0.01
5	509.7	2.432	0.12	510.4	2.429	0.14	527.8	2.349	0.05
1	675.5	1.835	0.31	670.9	1.848	0.29	678.2	1.828	0.23
2	622.9	1.990	0.16	621.8	1.994	0.17	659.4	1.880	0.05
3	601.9	2.060	0.01	561.5	2.208	0.00	607.7	2.040	0.17
4	519.3	2.388	0.05	522.1	2.375	0.04	529.3	2.342	0.02
5	511.6	2.423	0.12	513.7	2.414	0.12	520.8	2.381	0.03
1	677.8	1.829	0.31	669.1	1.853	0.28	697.8	1.777	0.05
2	633.8	1.956	0.01	619.3	2.002	0.16	672.4	1.844	0.24
3	621.8	1.994	0.16	542.1	2.287	0.00	605.7	2.047	0.16
4	520.7	2.381	0.05	519.7	2.386	0.04	547.2	2.266	0.00
5	512.3	2.420	0.11	506.7	2.447	0.12	521.8	2.376	0.05
1	671.6	1.846	0.31	670.7	1.849	0.28	718.5	1.726	0.02
2	619.0	2.003	0.17	618.9	2.003	0.17	677.2	1.831	0.27
3	581.1	2.134	0.00	533.2	2.325	0.00	607.1	2.042	0.16
4	521.7	2.377	0.06	520.9	2.380	0.04	558.1	2.222	0.00
5	509.8	2.432	0.14	509.7	2.432	0.12	522.9	2.371	0.05
1	671.2	1.847	0.31	668.2	1.855	0.29	695.4	1.783	0.06
2	623.6	1.988	0.17	623.4	1.989	0.18	672.3	1.844	0.23
3	554.3	2.237	0.00	543.9	2.280	0.00	607.7	2.040	0.16
4	517.3	2.397	0.05	518.9	2.389	0.05	543.4	2.282	0.00
5	511.2	2.425	0.12	513.2	2.416	0.11	520.4	2.382	0.05
1	678.0	1.829	0.31	676.5	1.833	0.27	715.5	1.733	0.02
2	625.3	1.983	0.12	658.1	1.884	0.02	673.6	1.841	0.26
3	618.7	2.004	0.05	613.0	2.023	0.17	605.4	2.048	0.16
4	520.5	2.382	0.05	529.6	2.341	0.01	558.2	2.221	0.00
5	515.1	2.407	0.11	522.7	2.372	0.03	521.3	2.378	0.05
1	676.9	1.832	0.31	670.0	1.851	0.28	674.4	1.838	0.28
2	618.9	2.003	0.17	617.9	2.007	0.17	620.1	1.999	0.02
3	592.4	2.093	0.00	591.9	2.095	0.00	612.1	2.026	0.15
4	520.7	2.381	0.06	518.9	2.389	0.04	523.6	2.368	0.05
5	509.7	2.432	0.13	511.9	2.422	0.12	508.3	2.439	0.12
1	677.7	1.829	0.29	667.0	1.859	0.28	769.1	1.612	0.00
2	638.3	1.942	0.01	619.6	2.001	0.16	680.3	1.822	0.30
3	619.5	2.001	0.16	532.7	2.327	0.00	618.1	2.006	0.17
4	523.2	2.370	0.04	519.7	2.386	0.05	589.4	2.104	0.00
5	509.2	2.435	0.09	508.9	2.436	0.10	524.4	2.364	0.05
1	682.2	1.817	0.22	669.9	1.851	0.28	763.6	1.624	0.00
2	663.3	1.869	0.09	612.1	2.026	0.16	680.4	1.822	0.30
3	618.1	2.006	0.17	585.1	2.119	0.00	623.0	1.990	0.17
4	530.6	2.337	0.01	520.5	2.382	0.05	585.1	2.119	0.00
5	517.9	2.394	0.04	506.4	2.448	0.12	521.6	2.377	0.05
1	678.6	1.827	0.30	676.4	1.833	0.23	676.2	1.834	0.28
2	631.7	1.963	0.01	662.6	1.871	0.06	666.4	1.861	0.02
3	612.3	2.025	0.16	609.3	2.035	0.16	624.8	1.984	0.17
4	522.1	2.375	0.05	534.9	2.318	0.00	528.4	2.346	0.00
5	511.2	2.425	0.12	520.7	2.381	0.05	519.1	2.388	0.05
1	683.7	1.813	0.28	674.5	1.838	0.11	785.8	1.578	0.00
2	619.2	2.002	0.03	662.0	1.873	0.16	673.8	1.840	0.29
3	603.7	2.054	0.14	610.8	2.030	0.16	619.9	2.000	0.17
4	523.4	2.369	0.04	542.1	2.287	0.00	596.2	2.080	0.00
5	503.3	2.463	0.13	519.9	2.385	0.05	518.4	2.392	0.04
1	678.0	1.829	0.30	671.2	1.847	0.28	755.1	1.642	0.01
2	616.0	2.013	0.16	615.6	2.014	0.02	677.9	1.829	0.30
3	588.9	2.105	0.00	610.0	2.033	0.14	617.3	2.008	0.16
4	521.1	2.379	0.05	522.1	2.375	0.05	584.1	2.123	0.00
5	509.8	2.432	0.12	507.3	2.444	0.13	522.7	2.372	0.07
1	680.0	1.823	0.30	670.2	1.850	0.29	670.5	1.849	0.30
2	613.1	2.022	0.16	615.7	2.014	0.17	622.5	1.992	0.16
3	598.9	2.070	0.01	582.4	2.129	0.00	605.1	2.049	0.00
4	520.5	2.382	0.05	520.4	2.382	0.05	518.4	2.392	0.07
5	511.2	2.425	0.13	508.0	2.441	0.11	513.9	2.413	0.11

1	675.6	1.835	0.31	672.0	1.845	0.28	709.8	1.747	0.02
2	616.1	2.012	0.16	638.5	1.942	0.01	674.6	1.838	0.28
3	564.2	2.198	0.00	609.8	2.033	0.16	616.0	2.013	0.17
4	521.5	2.377	0.06	522.1	2.375	0.04	556.4	2.228	0.00
5	510.6	2.428	0.12	515.1	2.407	0.01	522.0	2.375	0.06
1	675.1	1.837	0.31	672.5	1.844	0.22	679.6	1.824	0.25
2	617.4	2.008	0.17	659.4	1.880	0.07	665.0	1.864	0.05
3	539.0	2.300	0.00	611.3	2.028	0.16	621.6	1.995	0.16
4	520.7	2.381	0.06	535.2	2.317	0.00	529.7	2.341	0.01
5	509.5	2.433	0.12	518.3	2.392	0.05	520.8	2.381	0.05
1	675.9	1.834	0.31	676.6	1.832	0.28	673.6	1.841	0.30
2	616.3	2.012	0.16	619.4	2.002	0.17	621.3	1.996	0.03
3	562.8	2.203	0.00	607.2	2.042	0.00	616.6	2.011	0.13
4	517.0	2.398	0.06	524.2	2.365	0.05	519.2	2.388	0.07
5	507.2	2.444	0.12	510.9	2.427	0.12	511.5	2.424	0.13
1	677.0	1.831	0.31	674.3	1.839	0.28	733.1	1.691	0.01
2	612.7	2.024	0.16	630.5	1.966	0.00	675.7	1.835	0.29
3	565.9	2.191	0.00	619.2	2.002	0.17	612.9	2.023	0.16
4	520.2	2.383	0.06	520.4	2.382	0.04	570.8	2.172	0.00
5	510.9	2.427	0.13	511.5	2.424	0.13	520.9	2.380	0.07
1	676.0	1.834	0.31	675.8	1.835	0.28	668.4	1.855	0.30
2	616.6	2.011	0.17	620.5	1.998	0.05	621.9	1.994	0.16
3	531.1	2.334	0.00	617.6	2.008	0.12	581.3	2.133	0.00
4	518.6	2.391	0.06	522.0	2.375	0.05	515.3	2.406	0.07
5	507.7	2.442	0.12	511.0	2.426	0.14	512.0	2.422	0.12
1	674.7	1.838	0.31	677.3	1.831	0.28	710.5	1.745	0.01
2	618.9	2.003	0.17	623.0	1.990	0.17	672.4	1.844	0.29
3	527.5	2.350	0.00	615.6	2.014	0.01	614.3	2.018	0.16
4	518.9	2.389	0.06	522.6	2.372	0.05	560.4	2.212	0.00
5	510.7	2.428	0.11	511.6	2.423	0.13	520.0	2.384	0.07

Table S8. QM(TD-DFT)/MM excitation energies and corresponding electrochromic shifts (energies are in eV, shifts are in meV) of Chl$_{D1}$ and Pheo$_{D1}$ in each PsbA variant, computed on the QM/MM optimized geometries of **Chl$_{D1}$–Pheo$_{D1}$** embedded within the protein matrix for "crystal-like" snapshot 1.

**PsbA1**																		
**STATE**	**WT (no-H)**	**Gln130**		**Tyr147**		**Ser153**		**Phe158**		**Met172**		**Cys144**		**Cys212**		**WT (with H)**	**A1(Gln/Glu(H))**	
**Qy (Chl$_{D1}$)**	1.810	1.816	-6	1.809	1	1.822	-12	1.815	-5	1.819	-9	1.808	2	1.809	1	1.806	1.812	6
**Qx (Chl$_{D1}$)**	2.355	2.361	-6	2.354	1	2.365	-10	2.358	-3	2.355	0	2.352	3	2.354	1	2.346	2.356	10
**Qy (Pheo$_{D1}$)**	2.045	2.033	12	2.032	13	2.046	-1	2.046	-1	2.046	-1	2.046	-1	2.047	-2	2.048	2.042	-6
**Qx (Pheo$_{D1}$)**	2.445	2.431	14	2.445	0	2.444	1	2.445	0	2.445	0	2.443	2	2.446	-1	2.447	2.442	-5
**CT1**	1.911	2.000	-89	1.931	-20	1.980	-69	1.907	4	1.918	-7	1.900	11	1.907	4	1.885	1.909	24
**CT2**	2.413	2.503	-90	2.432	-19	2.478	-65	2.409	4	2.411	2	2.404	9	2.410	3	2.392	2.411	19
**PsbA2**																		
**STATE**	**WT (no-H)**	**Glh130**		**Phe147**		**Ala153**		**Leu158**		**Leu172**		**Pro144**		**Ala212**		**WT (with H)**	**A2(Glu(H)/Gln)**	
**Qy (Chl$_{D1}$)**	1.818	1.823	-5	1.819	-1	1.826	-8	1.820	-2	1.81	8	1.816	2	1.818	0	1.816	1.827	-11
**Qx (Chl$_{D1}$)**	2.346	2.352	-6	2.347	-1	2.353	-7	2.349	-3	2.338	8	2.342	4	2.347	-1	2.343	2.355	-12
**Qy (Pheo$_{D1}$)**	2.037	2.011	26	2.028	9	2.042	-5	2.038	-1	2.038	-1	2.039	-2	2.038	-1	2.029	2.03	-1
**Qx (Pheo$_{D1}$)**	2.433	2.426	7	2.437	-4	2.438	-5	2.433	0	2.44	-7	2.439	-6	2.435	-2	2.431	2.436	-5
**CT1**	1.955	2.046	-91	1.975	-20	2.007	-52	1.954	1	1.918	37	1.934	21	1.959	-4	1.932	1.938	-6
**CT2**	2.444	2.517	-73	2.461	-17	2.493	-49	2.444	0	2.405	39	2.417	27	2.447	-3	2.415	2.421	-6
**PsbA3**																		
**STATE**	**WT (no-H)**	**Glh130**		**Tyr147**		**Ala153**		**Phe158**		**Met172**		**Cys144**		**Ser212**		**WT (with H)**	**A3(Glu(H)/Gln)**	
**Qy (Chl$_{D1}$)**	1.819	1.843	-24	1.829	-10	1.832	-13	1.824	-5	1.83	-11	1.818	1	1.818	1	1.819	1.808	11
**Qx (Chl$_{D1}$)**	2.368	2.376	-8	2.369	-1	2.373	-5	2.373	-5	2.374	-6	2.367	1	2.368	0	2.368	2.345	23
**Qy (Pheo$_{D1}$)**	2.033	2.016	17	2.02	13	2.033	0	2.034	-1	2.033	0	2.035	-2	2.035	-2	2.027	2.034	-7
**Qx (Pheo$_{D1}$)**	2.434	2.423	11	2.429	5	2.434	0	2.434	0	2.434	0	2.432	2	2.434	0	2.427	2.443	-16
**CT1**	1.707	1.779	-72	1.758	-51	1.761	-54	1.703	4	1.714	-7	1.688	19	1.701	6	1.701	1.889	-188
**CT2**	2.229	2.314	-85	2.286	-57	2.285	-56	2.223	6	2.223	6	2.209	20	2.223	6	2.223	2.393	-170

SI References

1. Y. Umena, K. Kawakami, J.-R. Shen, N. Kamiya, Crystal structure of oxygen-evolving photosystem II at a resolution of 1.9 Å. *Nature* **473**, 55-60 (2011).
2. Y. Nakajima *et al.*, Crystal structures of photosystem II from a cyanobacterium expressing psbA2 in comparison to psbA3 reveal differences in the D1 subunit. *J. Biol. Chem.* **298**, 102668 (2022).
3. B. Loll *et al.*, Modeling of variant copies of subunit D1 in the structure of photosystem II from Thermosynechococcus elongatus. *Biol. Chem.* **389**, 609-617 (2008).
4. S. Schott-Verdugo, H. Gohlke, PACKMOL-Memgen: A Simple-To-Use, Generalized Workflow for Membrane-Protein–Lipid-Bilayer System Building. *J. Chem. Inf. Model.* **59**, 2522-2528 (2019).
5. L. Martínez, R. Andrade, E. G. Birgin, J. M. Martínez, PACKMOL: A package for building initial configurations for molecular dynamics simulations. *J. Comput. Chem.* **30**, 2157-2164 (2009).
6. W. L. Jorgensen, J. Chandrasekhar, J. D. Madura, R. W. Impey, M. L. Klein, Comparison of simple potential functions for simulating liquid water. *J. Chem. Phys.* **79**, 926-935 (1983).
7. X. He, V. H. Man, W. Yang, T.-S. Lee, J. Wang, A fast and high-quality charge model for the next generation general AMBER force field. *J. Chem. Phys.* **153**, 114502 (2020).
8. R. Salomon-Ferrer, D. A. Case, R. C. Walker, An overview of the Amber biomolecular simulation package. *WIREs Computational Molecular Science* **3**, 198-210 (2013).
9. J. Wang, R. M. Wolf, J. W. Caldwell, P. A. Kollman, D. A. Case, Development and testing of a general amber force field. *J. Comput. Chem.* **25**, 1157-1174 (2004).
10. Y. Duan *et al.*, A point-charge force field for molecular mechanics simulations of proteins based on condensed-phase quantum mechanical calculations. *J. Comput. Chem.* **24**, 1999-2012 (2003).
11. W. D. Cornell *et al.*, A Second Generation Force Field for the Simulation of Proteins, Nucleic Acids, and Organic Molecules. *J. Am. Chem. Soc.* **117**, 5179-5197 (1995).
12. C. I. Bayly, P. Cieplak, W. Cornell, P. A. Kollman, A well-behaved electrostatic potential based method using charge restraints for deriving atomic charges: the RESP model. *J. Phys. Chem.* **97**, 10269-10280 (1993).
13. C. J. Dickson *et al.*, Lipid14: The Amber Lipid Force Field. *J. Chem. Theory Comput.* **10**, 865-879 (2014).
14. A. Sirohiwal, D. A. Pantazis, Reaction Center Excitation in Photosystem II: From Multiscale Modeling to Functional Principles. *Acc. Chem. Res.* **56**, 2921-2932 (2023).
15. A. Sirohiwal, F. Neese, D. A. Pantazis, Protein Matrix Control of Reaction Center Excitation in Photosystem II. *J. Am. Chem. Soc.* **142**, 18174-18190 (2020).
16. N. Sakashita, H. C. Watanabe, T. Ikeda, K. Saito, H. Ishikita, Origins of Water Molecules in the Photosystem II Crystal Structure. *Biochemistry* **56**, 3049-3057 (2017).
17. F. Guerra, M. Siemers, C. Mielack, A.-N. Bondar, Dynamics of Long-Distance Hydrogen-Bond Networks in Photosystem II. *J. Phys. Chem. B* **122**, 4625-4641 (2018).
18. K. Saito, A. W. Rutherford, H. Ishikita, Mechanism of proton-coupled quinone reduction in Photosystem II. *Proc. Natl. Acad. Sci. U. S. A.* **110**, 954-959 (2013).
19. M. Ceccarelli, P. Procacci, M. Marchi, Anab initio force field for the cofactors of bacterial photosynthesis. *J. Comput. Chem.* **24**, 129-142 (2003).
20. F. Guerra, S. Adam, A.-N. Bondar, Revised force-field parameters for chlorophyll-a, pheophytin-a and plastoquinone-9. *J. Mol. Graphics Modell.* **58**, 30-39 (2015).
21. R. J. Loncharich, B. R. Brooks, R. W. Pastor, Langevin dynamics of peptides: The frictional dependence of isomerization rates of N-acetylalanyl-N′-methylamide. *Biopolymers* **32**, 523-535 (1992).
22. H. J. C. Berendsen, J. P. M. Postma, W. F. van Gunsteren, A. DiNola, J. R. Haak, Molecular dynamics with coupling to an external bath. *J. Chem. Phys.* **81**, 3684-3690 (1984).
23. J.-P. Ryckaert, G. Ciccotti, H. J. C. Berendsen, Numerical integration of the cartesian equations of motion of a system with constraints: molecular dynamics of n-alkanes. *J. Comput. Phys.* **23**, 327-341 (1977).
24. R. Salomon-Ferrer, A. W. Götz, D. Poole, S. Le Grand, R. C. Walker, Routine Microsecond Molecular Dynamics Simulations with AMBER on GPUs. 2. Explicit Solvent Particle Mesh Ewald. *J. Chem. Theory Comput.* **9**, 3878-3888 (2013).
25. P. Li, B. P. Roberts, D. K. Chakravorty, K. M. Merz, Jr., Rational Design of Particle Mesh Ewald Compatible Lennard-Jones Parameters for +2 Metal Cations in Explicit Solvent. *J. Chem. Theory Comput.* **9**, 2733-2748 (2013).
26. S. Le Grand, A. W. Götz, R. C. Walker, SPFP: Speed without compromise—A mixed precision model for GPU accelerated molecular dynamics simulations. *Comput. Phys. Commun.* **184**, 374-380 (2013).
27. U. Essmann *et al.*, A smooth particle mesh Ewald method. *J. Chem. Phys.* **103**, 8577-8593 (1995).
28. D. A. Case *et al.*, AmberTools. *J. Chem. Inf. Model.* **63**, 6183-6191 (2023).
29. D. R. Roe, T. E. Cheatham Iii, Parallelization of CPPTRAJ enables large scale analysis of molecular dynamics trajectory data. *J. Comput. Chem.* **39**, 2110-2117 (2018).
30. D. R. Roe, T. E. Cheatham, III, PTRAJ and CPPTRAJ: Software for Processing and Analysis of Molecular Dynamics Trajectory Data. *J. Chem. Theory Comput.* **9**, 3084-3095 (2013).
31. W. Humphrey, A. Dalke, K. Schulten, VMD: Visual molecular dynamics. *J. Mol. Graphics* **14**, 33-38 (1996).
32. W. L. DeLano, The PyMOL molecular graphics system. http://www.pymol.org/ (2002).

33. P. A. Kollman *et al.*, Calculating Structures and Free Energies of Complex Molecules: Combining Molecular Mechanics and Continuum Models. *Acc. Chem. Res.* **33**, 889-897 (2000).
34. J. P. Perdew, K. Burke, M. Ernzerhof, Generalized Gradient Approximation Made Simple. *Phys. Rev. Lett.* **77**, 3865-3868 (1996).
35. F. Weigend, R. Ahlrichs, Balanced basis sets of split valence, triple zeta valence and quadruple zeta valence quality for H to Rn: Design and assessment of accuracy. *Phys. Chem. Chem. Phys.* **7**, 3297-3305 (2005).
36. S. Grimme, J. Antony, S. Ehrlich, H. Krieg, A consistent and accurate ab initio parametrization of density functional dispersion correction (DFT-D) for the 94 elements H-Pu. *J. Chem. Phys.* **132**, 154104 (2010).
37. S. Grimme, S. Ehrlich, L. Goerigk, Effect of the damping function in dispersion corrected density functional theory. *J. Comput. Chem.* **32**, 1456-1465 (2011).
38. F. Neese, An improvement of the resolution of the identity approximation for the formation of the Coulomb matrix. *J. Comput. Chem.* **24**, 1740-1747 (2003).
39. F. Weigend, Accurate Coulomb-fitting basis sets for H to Rn. *Phys. Chem. Chem. Phys.* **8**, 1057-1065 (2006).
40. N. Mardirossian, M. Head-Gordon, ωB97X-V: A 10-parameter, range-separated hybrid, generalized gradient approximation density functional with nonlocal correlation, designed by a survival-of-the-fittest strategy. *Phys. Chem. Chem. Phys.* **16**, 9904 (2014).
41. A. Sirohiwal, F. Neese, D. A. Pantazis, How Can We Predict Accurate Electrochromic Shifts for Biochromophores? A Case Study on the Photosynthetic Reaction Center. *J. Chem. Theory Comput.* **17**, 1858-1873 (2021).
42. F. Neese, F. Wennmohs, A. Hansen, U. Becker, Efficient, approximate and parallel Hartree–Fock and hybrid DFT calculations. A 'chain-of-spheres' algorithm for the Hartree–Fock exchange. *Chem. Phys.* **356**, 98-109 (2009).
43. R. L. Martin, Natural transition orbitals. *J. Chem. Phys.* **118**, 4775-4777 (2003).
44. F. Neese, Software update: The ORCA program system—Version 5.0. *WIREs Computational Molecular Science* **12**, e1606 (2022).
45. F. Neese, F. Wennmohs, U. Becker, C. Riplinger, The ORCA quantum chemistry program package. *J. Chem. Phys.* **152**, 224108 (2020).
46. C. G. Chen, A. N. Nardi, A. Amadei, M. D'Abramo, PyMM: An Open-Source Python Program for QM/MM Simulations Based on the Perturbed Matrix Method. *J. Chem. Theory Comput.* **19**, 33-41 (2023).
47. A. Amadei, M. Aschi, Theoretical-computational modeling of charge transfer and intersystem crossing reactions in complex chemical systems. *RSC Adv.* **8**, 27900-27918 (2018).
48. M. Aschi, R. Spezia, A. Di Nola, A. Amadei, A first-principles method to model perturbed electronic wavefunctions: the effect of an external homogeneous electric field. *Chem. Phys. Lett.* **344**, 374-380 (2001).
49. E. Jurrus *et al.*, Improvements to the APBS biomolecular solvation software suite. *Protein Sci.* **27**, 112-128 (2018).
